# A family of genus‐specific RNAs in tandem with DNA‐binding proteins control expression of the *badA* major virulence factor gene in *Bartonella henselae*

**DOI:** 10.1002/mbo3.420

**Published:** 2016-10-28

**Authors:** Nhan Tu, Ronan K. Carroll, Andy Weiss, Lindsey N. Shaw, Gael Nicolas, Sarah Thomas, Amorce Lima, Udoka Okaro, Burt Anderson

**Affiliations:** ^1^ Department of Molecular MedicineMorsani College of MedicineUniversity of South FloridaTampaFLUSA; ^2^ Department of Biological SciencesOhio UniversityAthensOHUSA; ^3^ Department of Cellular, Molecular and MicrobiologyCollege of Arts and SciencesUniversity of South FloridaTampaFLUSA

**Keywords:** *Bartonella henselae*, gene regulation, sRNA, transcriptional regulator, trimeric autotransporter adhesin, xenobiotic response element

## Abstract

*Bartonella henselae* is a gram‐negative zoonotic bacterium that causes infections in humans including endocarditis and bacillary angiomatosis. *B. henselae* has been shown to grow as large aggregates and form biofilms in vitro. The aggregative growth and the angiogenic host response requires the trimeric autotransporter adhesin BadA. We examined the transcriptome of the Houston‐1 strain of *B. henselae* using RNA‐seq revealing nine novel, highly‐expressed intergenic transcripts (*Bartonella* regulatory transcript, Brt1‐9). The Brt family of RNAs is unique to the genus *Bartonella* and ranges from 194 to 203 nucleotides with high homology and stable predicted secondary structures. Immediately downstream of each of the nine RNA genes is a helix‐turn‐helix DNA‐binding protein (transcriptional regulatory protein, Trp1‐9) that is poorly transcribed under the growth conditions used for RNA‐seq. Using knockdown or overexpressing strains, we show a role of both the Brt1 and Trp1 in the regulation of *badA* and also in biofilm formation. Based on these data, we hypothesize that Brt1 is a *trans*‐acting sRNA that also serves as a *cis*‐acting riboswitch to control the expression of *badA*. This family of RNAs together with the downstream Trp DNA‐binding proteins represents a novel coordinated regulatory circuit controlling expression of virulence‐associated genes in the bartonellae.

## 1 Introduction

The genus *Bartonella* are gram‐negative, arthropod vector‐borne facultative intracellular bacteria that infect a wide range of hosts (Anderson & Neuman, 1997). While humans are the reservoir hosts for *Bartonella bacilliformis* and *Bartonella quintana*, cats are the natural host for *B. henselae* in which they usually cause an asymptomatic intraerythrocytic infection. The organism is transmitted between cats by the cat flea (*Ctenocephalides felis*), and from infected cats to humans by scratching. Immunocompetent individuals infected with *B. henselae* typically suffer from cat scratch disease (CSD), which is a self‐limiting infection. In contrast, immunosuppressed individuals infected with *B. henselae* can develop systemic infections including bacillary angiomatosis and bacillary peliosis, characterized by vasoproliferative tumor‐like lesions on the skin and liver, respectively (Anderson & Neuman, 1997). These lesions are a result of proliferation of vascular endothelial cells found in close association with aggregates of *B. henselae* (Dehio, 2005). In addition, *B. henselae* is a common cause of fever of unknown origin and endocarditis and has been detected in vegetative masses on infected heart valves from patients with culture‐negative endocarditis (Edouard, Nabet, Lepidi, Fournier, & Raoult, 2015). It has also been reported to grow as large aggregates in vitro and to form biofilms (Kyme, Dillon, & Iredell, 2003).

The induction of this unique angiogenic host response as well as the aggregative growth of this bacterium is known to involve the high‐molecular weight trimeric autotransporter adhesin (TAA) BadA. BadA is a large surface‐exposed filament‐shaped protein of 3036 amino acids and a length of 240 nm (Muller et al., 2011). The role of BadA in *B. henselae* pathogenesis has been studied extensively in vitro and is critical for autoagglutination, adhesion to host cells, and extracellular matrix proteins such as collagen, inhibition of phagocytosis, and induction of angiogenesis (Riess et al., 2004). In vitro studies show that the expression of *badA* correlates with a proangiogenic cell response via activation of HIF‐1 and NF‐kB, and via the secretion of vascular endothelial growth factor (VEGF) and IL‐8 (Kempf et al., 2001, 2005; McCord, Burgess, Whaley, & Anderson, 2005; McCord, Resto‐Ruiz, & Anderson, 2006; Riess et al., 2004). Despite the critical role of BadA in the pathogenesis of *B. henselae*, very little is known about how *badA* is regulated and why some strains express high levels of *badA* and have an autoaggregative phenotype, while others express little if any *badA* and are not autoaggregative (Riess, Raddatz, Linke, Schafer, & Kempf, 2007).

As a zoonotic bacterium, *B. henselae* must be able to adapt to very diverse environments such as the cat flea vector where the temperature is low and, after a blood meal, hemin reaches toxic levels. In contrast, the vertebrate host temperature is higher and hemin availability is limited. Very little is known about how *B. henselae* adapts its gene expression profiles to efficiently transition from the conditions of the cat flea vector to the mammalian hosts. Reports have proposed a role for several different regulatory proteins in various aspects of host adaptation in *B. henselae* and *B. quintana*. (Battisti, Sappington, Smitherman, Parrow, & Minnick, 2006; Quebatte, Dick, Kaever, Schmidt, & Dehio, 2013; Quebatte et al., 2010; Roden, Wells, Chomel, Kasten, & Koehler, 2012). Recently, a novel alternate sigma factor activation mechanism has been shown to induce a general stress response in *B. quintana* (Abromaitis & Koehler, 2013). This general stress response was shown to regulate genes in response to temperature that corresponds to a shift from the vector to humans and vice versa (Abromaitis et al., 2013). This same general stress response has been described in *B. henselae* and shown to play a role in regulating *badA* (Tu, Lima, Bandeali, & Anderson, 2016). However, a role of RNA in gene regulation and host adaptation in *Bartonella* remains unexplored despite the well‐described role of RNAs in rapidly modulating gene expression profiles in other bacteria.

RNAs can regulate expression in bacteria by acting in *trans* on genes distal to the RNA coding region or in *cis* by acting on adjacent genes either downstream or on the opposite strand. Small regulatory RNAs (sRNAs) function by binding target mRNAs, binding proteins, or by targeting and degrading specific mRNAs (see [Michaux, Verneuil, Hartke, & Giard, 2014] for review of sRNA functions). sRNAs may be *cis*‐acting or *trans*‐acting and their biological roles have been shown to include regulation of metabolism, growth, adaptation to stress, or pathogenesis (Gong et al., 2011; Koo, Alleyne, Schiano, Jafari, & Lathem, 2011; Toledo‐Arana, Repoila, & Cossart, 2007; Vogel, 2009; Waters & Storz, 2009). In contrast, *cis*‐acting RNAs including riboswitches involve structured domains found on the noncoding regions of the mRNA being regulated. Binding of ligands or metabolites is the primary mechanism resulting in the formation of functional domains in riboswitches. Thus, the presence and concentration of these ligands are the triggers that activate the riboswitch. This sensing and response to ligands typically results in changes in mRNA secondary structure, resulting in transcriptional read‐through, altered stability of the mRNA, or changes in translation efficiency thereby effecting modified gene expression (see (Winkler & Breaker, 2005) for review of riboswitches).

Here, we describe a highly transcribed family of nine small RNAs (*Bartonella* regulatory transcripts, Brt1‐9) that are unique to *Bartonella* species. All nine of these RNAs are immediately upstream of a coding region for a protein that is annotated as a helix‐turn‐helix DNA‐binding protein/transcriptional regulator (transcriptional regulatory protein, Trp1‐9). We show a role for the most highly transcribed member of this RNA family, Brt1, in regulating *badA* and we further show that the cognate Trp1 positively controls *badA* expression. We hypothesize that the Brt family of RNAs are capable of functioning as a two‐tiered system including both *cis*‐acting and *trans*‐acting roles to coordinate regulation of *badA*, which is responsible for autoaggregation, host cell attachment, the proangiogenic host response, and biofilm formation by *B. henselae*.

## 2 Experimental Procedures

### 2.1 Bacterial strains and growth conditions

The Houston‐1 strain of *B. henselae* which was isolated from an HIV‐infected patient (Regnery et al., 1992) was used for all experiments and was cultured on heart infusion agar supplemented with 1% bovine hemoglobin (chocolate agar) and incubated for 3–4 days at 37°C in the presence of 5% CO_2_. For some experiments, bacteria were grown in Schneider's liquid media (Sigma Aldrich, S9895) supplemented with 10% fetal bovine serum for 3–4 days at 37°C in the presence of 5% CO_2_ as previously described (Riess et al., 2008). For experiments using strains harboring pNS2‐derived plasmids, the culture medium was supplemented with 50 μg/ml kanamycin (Gillaspie et al., 2009). The construction of a nonpolar in‐frame deletion mutant of *badA* (BH01510) has been previously described (Lima, Cha, Amin, Smith, & Anderson, 2014). The ∆*badA* mutant served as a control for experiments examining *badA* expression and biofilm formation. All *B. henselae* strains used in this study are described in Table 2. Growth curves indicate that there are no growth defects for the strains used in this study (Figure S2). All manipulations of *B. henselae* have been approved by the USF Institutional Biosafety Committee.

### 2.2 RNA isolation

*B. henselae* Houston‐1 was cultured in Schneider's liquid media at 37°C, 5% CO_2_ for 72 hr, and collected by centrifugation for RNA extraction. The bacteria were exposed to RNAProtect Cell Reagent (Qiagen, 76506) and pelleted and frozen at −80°C. The pellets were thawed at 4°C before RNA isolation. Total RNA was extracted using the RNeasy Mini Kit (Qiagen, 74104). Following extraction, RNA was treated with DNase (Thermo Fisher Scientific, AM1907). RNA quality was analyzed using the Agilent 2100 Bioanalyzer.

### 2.3 RNA‐seq

To remove ribosomal RNA (rRNA) for RNA‐Seq, Oligo Magbeads from the MICROBExpress Kit (Thermo Fisher Scientific, AM1905) that hybridize with the 16S and 23S rRNAs were used. A quantity of 3.4 μg of RNA was used for rRNA removal. The RNA sample was analyzed on the Bioanalyzer to monitor removal of rRNAs. The subsequent RNA processing was done using the Ion Total RNA‐Seq Kit v2 (Thermo Fisher Scientific, 4475936). The enriched RNA was fragmented with RNase III and run on the Bioanalyzer to analyze the size of the fragmented RNA. The fragmented RNA was then reverse transcribed into cDNA and used as a template for sequencing using an Ion Torrent Personal Genome Machine (Thermo Fisher Scientific). The sequenced transcriptome was aligned to the *B. henselae* Houston‐1 reference genome from NCBI (Alsmark et al., 2004). Data analysis was carried out using the CLC Genomics Workbench platform (CLC bio).

### 2.4 Northern blot

A 29 base oligonucleotide probe (Brtprobe, Table S6) was designed to base‐pair to a conserved sequence found in all nine Brts. For detecting antisense Brt1 RNA, a second probe which hybridized to RNA transcribed from the opposite (antisense) strand was used (asBrtprobe, Table S6). The probe was labeled with [γ‐^32^P]ATP using T4 polynucleotide kinase (Promega, M4101). Unincorporated [γ‐^32^P]ATP was then removed using a QIAquick Nucleotide Removal Kit (Qiagen, 28304). *B. henselae* strains were cultured in Schneider's medium or Schneider's medium with 50 μg/ml kanamycin for 72 hr and total RNA was isolated with the RNeasy Mini Kit and treated with Turbo DNase. Four microgram of DNase‐treated total RNA was separated on a 10% denaturing polyacrylamide mini gel. The RNA was transferred to an Immobilon‐Ny^+^ Membrane (Millipore). Following transfer, the RNA was UV cross‐linked to the membrane. Prior to hybridization, the membrane was prehybridized in ULTRAhyb‐Oligo Buffer (Thermo Fisher Scientific, AM8663) at 42°C for 1 hr. The labeled probe was denatured by heating at 95°C for 5 min and added to the membrane to allow for hybridization overnight at 42°C in a hybridization oven. After hybridization, the membrane was washed for 15 min, each at 42°C sequentially with: 2X SSC, 1X SSC, and 0.5 SSC. The membrane was then exposed to a phosphorimager screen overnight. The image was developed using a Typhoon 9410 (GE HealthCare).

### 2.5 Bioinformatic analysis

*brt*1‐9 transcription start and stop sites were determined by RNA‐seq according to the start and end of the RNA transcripts. Brt nucleotide and Trp nucleotide and amino acid sequences were obtained from the *B. henselae* Houston‐1 genome sequence from NCBI (accession # BX897699). Nucleotide sequence alignment and percent identity was carried out using the multiple sequence alignment tool (MUSCLE) from EMBL‐EBI (Edgar, 2004). RNAfold was used to predict the secondary structures of individual Brt nucleotide sequences (Lorenz et al., 2011). The minimum free energy was calculated based on predicted secondary structure to indicate the stability of the predicted structure. Individual Brt nucleotide sequences were searched against the NCBI database to find related sequences in other bacteria species. The CLC Sequence View 6 (CLC bio) software was used to align Trp amino acid sequences and for phylogenetic analysis. Target predictions for individual Brts were carried out using RNApredator (Eggenhofer, Tafer, Stadler, & Hofacker, 2011) and IntaRNA (Busch, Richter, & Backofen, 2008; Wright et al., 2014).

### 2.6 Antisense Brt1 RNA knockdown plasmid construction

PCR primers (Brt1asR, Brt1asF, Table S6) were expected to amplify the entire Brt1 gene sequence from *B. henselae* Houston‐1 genomic DNA. The resulting amplicon was directionally ligated into the pNS2Trc plasmid using the *Bam*HI and *Xba*I restriction sites such that high levels of antisense RNA complementary to Brt1 would be transcribed from the Trc promoter in that plasmid. The plasmids were transformed into competent DH12S *E. coli* cells and selected for colonies harboring the plasmid with 50 μg/ml kanamycin. PCR was used to screen for successful ligation of the *brt*1 gene into the pNS2Trc plasmid in the expected orientation. After confirmation by sequencing, the plasmid was then electroporated into *B. henselae* Houston‐1 as previously described (Resto‐Ruiz, Sweger, Widen, Valkov, & Anderson, 2000), to produce strain *Bhh*72 (Table 2). qRT‐PCR with asBrt1 RNA‐specific primers showed high levels of asBrt1 in *Bhh72* compared to the control, confirming transcription of this antisense RNA (Figure 6a). For reasons that are unclear, the antisense RNA detected by northern blots migrated at approximately 200 nucleotides (Figure 6f, lane 2), smaller than the expected size of approximately 350 nucleotides.

### 2.7 Overexpression of Trp1 plasmid construction

PCR primers specific for the *trp*1 gene were designed to amplify the gene from genomic *B. henselae* Houston‐1 DNA from the second codon of the gene to beyond the stop codon (Trp1F, Trp1R, Figure S6). The resulting amplicon was ligated into the *Bam*HI and *Xba*I restriction sites of pNS2Trc to create an overexpressing Trp His‐tag fusion protein. The plasmids were transformed into competent DH12S *E. coli* cells and selected for colonies harboring the plasmid with 50 μg/ml kanamycin. PCR was used to screen for successful ligation of the *trp1* gene into the pNS2Trc plasmid in the intended orientation that would allow overexpression of the gene. The plasmids were then electroporated into *B. henselae* Houston‐1 as previously described (Resto‐Ruiz et al., 2000). It should be noted that the *B. henselae* genome does not contain the lactose repressor gene, and hence, the P_*trc*_ promoter of pNS2P_*Trc*_ is constitutive and not inducible in *B. henselae* (Gillaspie et al., 2009). Expression of 6XHis‐tagged Trp1 was confirmed in the resulting strain (*Bhh*73, Table 2) by western blot with mouse anti‐His tag antibody (Figure 5a).

### 2.8 qRT‐PCR of *B. henselae* genes

The antisense Brt1 strain and the overexpressing *trp1* strain of *B. henselae* were used to analyze the transcription of the *badA* gene. Bacteria were grown in Schneider's medium for 3 days and cells were collected by centrifugation and total RNA was extracted, treated with Turbo DNase (Thermo Fisher Scientific, AM1907), and converted into cDNA using the iScript cDNA synthesis kit (BioRad, 1708891). qRT‐PCR was performed in 25 μl reaction volumes that include 12.5 μl of the 2X Maxima SYBR Green/Fluorescein qPCR kit (Thermo Fisher Scientific, K0241), 300 nmol of each gene‐specific primer, and 2 μl cDNA. All reactions were performed in triplicate and 50S ribosomal protein L4 (*rplD*) was used as the reference gene for normalization. The qRT‐PCR cycling parameters included 95°C for 3 min, 40 cycles of 95°C for 10 s, and 60°C for 30 s, followed by 95°C for 45 s and 55°C for 1 min. Melting curve analysis was performed to confirm that no primer dimers were amplified. Results were analyzed using the comparative C_T_ method (Schmittgen & Livak, 2008). The primer pairs used for qRT‐PCR of the Brts, *badA*, and *trp1* are shown in Table S6.

### 2.9 Dot‐blot assay for BadA

To further determine if qRT‐PCR for transcription of the *badA* gene could be correlated with BadA protein production, we performed dot‐blots using rabbit polyvalent antibody to BadA (a generous gift from Dr. Volkhard Kempf). Since BadA is a surface trimeric autotransporter adhesin, it exists in *B. henselae* as a high‐molecular weight (>1 million daltons) fibrillar complexes (Riess et al., 2004). The individual subunits of BadA are approximately 330,000 daltons, making gel resolution and protein transfer to membranes inefficient and western blotting impractical. For this reason, dot‐blots of intact *B. henselae* cells grown for 3 days were performed. Two microliters containing approximately 1 × 10^6^ bacteria (standardized to 2.0 μg protein) in PBS was spotted onto a nitrocellulose filter. The membrane was then blocked with PBS +0.05% Tween 20 containing 5% skim milk. Antibody diluted 1:200 in PBS +5% skim milk was added and the filters were incubated for 2 hr at room temperature and washed four times in PBS +0.05% Tween 20. The resulting filters were incubated in goat anti‐rabbit conjugated with horseradish peroxidase (KPL, Gaithersburg, MD) diluted 1:10,000 in PBS +5% skim milk for 1 hr at room temperature. After three washes with PBS +0.05% Tween 20, the filters were incubated with ECL luminescent substrate (Thermo Scientific) for 1 min and detected using a Bio‐Rad Chemidoc XRS Imaging system.

### 2.10 Biofilm assay

A standard microplate adherence assay was used to measure *B. henselae* biofilm formation. This method is based on the standard crystal violet microplate assay for gram‐negative bacteria (O'Toole, 2011) that has been adapted for *B. henselae* (Kyme et al., 2003). Briefly, 5 × 10^6^ bacteria in a volume of 100 μl was added to each well of a 96‐well polystyrene plate (Costar, Corning, NY) and the plates were incubated for 3 days at 37°C with 5% CO_2_. After incubation, the plates were washed with water and subsequently stained with 125 μl of a 0.1% crystal violet solution for 15 min. After washing, the plates were allowed to dry for 3 hr and the stain was extracted with 125 μl 30% acetic acid for 15 min. The stain solution was transferred to a new 96‐well plate and the OD_550_ measured using a plate reader (BioTek, Winooski, VT).

### 2.11 Statistical analysis

The means ± standard errors were presented in the data. SigmaPlot software (Systat Software, San Jose, CA) was used for statistical analysis using the *t*‐test, with a *p*‐value of <.05 being considered statistically significant.

## 3 Results

### 3.1 RNA‐seq reveals nine unannotated highly transcribed short RNAs

**Figure 1:**
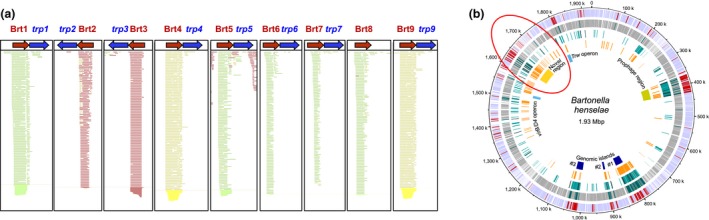
RNA‐seq analysis and genome location of the Brt family of RNAs. (a) RNA‐seq analysis showing the reads and direction of the nine Brt family RNAs. Green indicates transcription (left to right 5′ to 3′) in the direction depicted, red indicates transcription in the reverse direction (right to left as indicated), and yellow indicates regions with ambiguity (Brt4 and Brt9 have identical sequences) (b) Location of the nine Brt family of RNAs on the *Bartonella henselae* Houston‐1 genome. The red oval indicates the region of the genome containing all nine Brt RNA family members. Genome map reproduced from (Omasits et al., 2013) with permission.

The transcriptome of *B. henselae*, grown under standard growth conditions in Schneider's liquid medium at 37°C with 5% CO_2_ for 72 hr as previously described, (Riess et al., 2008) was examined using RNA‐Seq. Based on growth curves for the strains used in these studies, this correlates with late log‐phase growth. While the depth of coverage was low due to incomplete rRNA removal, it was not difficult to discern highly transcribed genes. Surprisingly, several small RNAs that did not map to know genes, were shown to be highly transcribed. Specifically, a group of nine short RNAs, that are not annotated in the published genome (Alsmark et al., 2004) or subsequently analyzed genome (Tatusova, Ciufo, Fedorov, O'Neill, & Tolstoy, 2014), were noted to be highly transcribed (Figure 1a). These RNAs varied in size from 194 to 203 nucleotides and were transcribed from noncoding regions of the *B. henselae* genome that did not map to or overlap any known genes. The RNAs were designated Brt1 through Brt9 (for *Bartonella* regulatory transcript) to identify each noncoding RNA. The genome location as well as the start and stop of transcription for all nine Brts can be found in Table S1. Specific details of the RNA‐seq results are found in File S1.

### 3.2 The Brt RNA loci cluster in a small region of the *B. henselae* genome

The nine Brt RNA loci were interspersed throughout a relatively small region (277 kb) of the *B. henselae* genome (Figure 1b, red oval). This region has been described as highly plastic and very likely contains several horizontally acquired mobile genetic elements (Lindroos et al., 2006). This region includes prophage genes as well as the pathogenicity island encoding the VirB type IV secretion system (Alsmark et al., 2004), which has been shown to play an important role in interaction with host cells and to play a role in the pathogenesis of *B. henselae* (Schulein et al., 2005). In a more recent study, the authors describe this region of the genome as a plastic “novel region” enriched in repeats with highly expressed genes for which proteins could not be detected. The authors of that study further suggested that this region contains highly transcribed genes that do not represent a bona fide protein‐coding ORF,(Omasits et al., 2013), an observation consistent with noncoding regulatory RNAs. Since the genome size of *B. henselae* is just over 1.9 Mbp, the region harboring the Brts represents only about 15% of the total genome. This clustering of the Brts taken together with their multiple copy number is strongly suggestive of a “hot spot” area of the genome undergoing active gene duplication or horizontal gene acquisition.

### 3.3 The nucleotide sequence of the nine Brt RNAs is highly conserved and are predicted to form complex secondary structures

**Figure 2:**
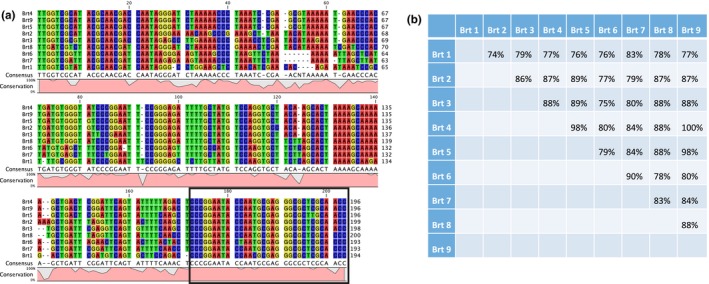
(a) Nucleotide sequence alignment, consensus sequence, and conservation of the nine Brt family RNA members. The most highly conserved region of the sequences is the 3′ end (black box). (b) Nucleotide sequence identity matrix for the nine Brts

**Figure 3:**
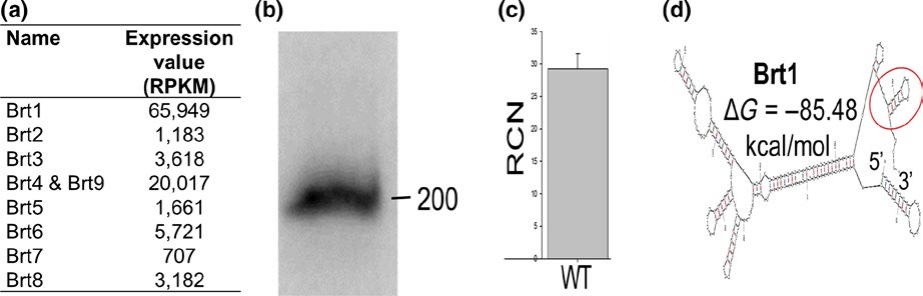
Analysis of Brt family of RNAs from wild‐type Houston‐1 strain of *Bartonella henselae* as determined by: (a) Expression values from RNA‐seq for Brt1‐9 (RPKM = reads/kilobase/million mapped reads). Since Brt4 and Brt9 are identical, their individual expression levels cannot be determined by RNA‐seq, their combined expression is depicted. (b) Northern blot with Brt family probe (c). qRT‐PCR with primers to conserved region of Brts showing relative copy number compared to reference *rplD* mRNA (d). The predicted secondary structure of Brt1 RNA with a potential terminator/riboswitch region (red oval) indicated

Nucleotide sequence alignment of Brt1‐Brt9 reveals a high level of conservation across the length of Brt1‐9 ranging from 74% to 100% (Figure 2, panel b) with a high degree of conservation in the middle of the RNAs and the highest sequence conservation at the 3′ terminus (Figure 2, panel a, black box). Each Brt was examined for predicted secondary structure and all nine were shown to have extensive possible base‐pairing with the most stable loop and stem structures varying from ΔG = −85.5 kcal/mol to ΔG = −56.3 kcal/mol (Figure S1). The predicted secondary structure for Brt1 includes a region which may serve as a potential transcription terminator or riboswitch region (Figure 3d, red oval). It was noted that each of the nine Brts could be seen to have considerable base‐pairing near these same central and 3′ terminal regions (Figure S1). Taken together, the highly transcribed Brts in the size range from 194 to 203 nucleotides with extensive predicted secondary structure located in noncoding regions of the genome are suggestive of small regulatory RNAs.

### 3.4 The Brt RNAs are genus‐specific, but vary in copy number among other *Bartonella* species

Bioinformatic analysis of the nine Brts indicated that they appear to be unique to the genus *Bartonella,* as no similar sequences were found in other alpha‐proteobacteria or any other bacteria. Each individual Brt nucleotide sequence was used as a query sequence against the NCBI genome database using the BLAST program and only *Bartonella* genomes were returned as results. The copy number of the Brt family varied greatly among *Bartonella* species and strains from nine copies found in *B. henselae* Houston‐1*,* seven each in the *B. henselae* cat isolates BM1374163 and BM1374165, 33 to 34 in *B. tribocorum*, 26 and one plasmid in *B. grahamii*, eight in *B. vinsonii* subsp. *berkhoffii*, and two or three in *B. schoenbuchensis*. Interestingly, no copies of the *brts* could be found in the genome of *B. bacilliformis* or *B. rochalimae* (Table 1). Thus, these novel RNAs appear to be genus‐specific, but are not found in all *Bartonella* species.

**Table 1:** Copy number of Brt family of RNAs and genome size among *Bartonella* species

Strain	Reservoir host	Genome size (Mb)	# of Brt RNAs
*Bartonella bacilliformis*	Human	1.45	0
*Bartonella rochalimae* ATCC BAA‐1498	Human	1.53	0
*Bartonella quintana* str. Toulouse	Human	1.58	2
*Bartonella quintana* str. RM‐11	Human	1.59	2
*Bartonella schoenbuchensis* str. MVT06	Deer	1.68	2
*Bartonella schoenbuchensis* R1	Deer	1.68	3
*Bartonella henselae* BM1374165	Cat	1.91	7
*Bartonella henselae* BM1374163	Cat	1.98	7
*Bartonella vinsonii subsp. Berkhoffii* str. Winnie	Dog	1.80	8
*Bartonella henselae* str. Houston‐1	Cat	1.93	9
*Bartonella grahamii* as4aup	Mouse	2.34	26 + 1 plasmid
*Bartonella tribocorum* str. BM1374166	Rat	2.62	33
*Bartonella tribocorum* CIP 105476	Rat	2.62	34

### 3.5 Brt1 is among the most highly transcribed regions of the *Bh* genome

Based on analysis of the quantitative data from the RNA‐seq, the RPKM (reads/kilobase/million mapped reads) were far higher (>10‐fold) for Brt1 than for any of the other Brt family of RNAs indicating that more copies of this RNA were present than for Brt2‐Brt9 combined (Figure 3a). In fact, *brt*1 was among the most highly transcribed regions of the *B. henselae* genome, with the number of reads among the highest four loci (excluding rRNAs) in the *Bh* transcriptome (File S1). The presence of Brt RNAs has also been confirmed by northern blot (Figure 3b), qRT‐PCR (Figure 3c), and in a previously published independent RNA‐seq analysis (Omasits et al., 2013). Accordingly, the Brt1 RNA was the focus of our subsequent studies based on this high level of transcription under the conditions tested with the expectation that modulating expression of *brt*1 would have the greatest effect on gene expression and phenotype if it is in fact a regulatory RNA.

### 3.6 All nine Brts are tandemly arranged on the *B. henselae* genome with a downstream transcriptional regulator gene

**Figure 4:**
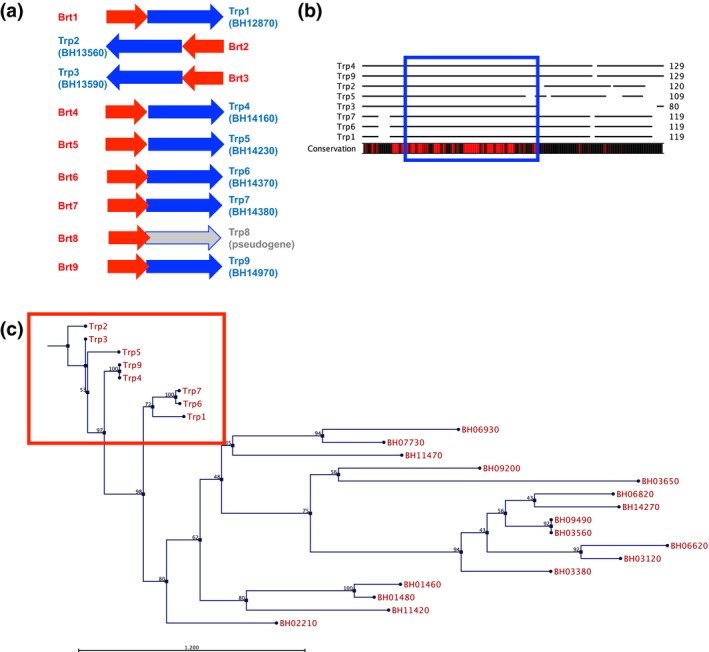
Trp‐encoding genes and proteins in *Bartonella henselae*. (a) Gene arrangement showing the *trp* genes in blue and corresponding Brt RNA coding regions in red. The genome locus for each *trp* is also shown. (b) Amino acid conservation with highly conserved areas indicated by red bar below the alignment. The helix‐turn‐helix DNA‐binding domain common to the xenobiotic response element family of proteins is boxed in blue. (c) Phylogenetic tree of the eight Trp family DNA‐binding proteins and 16 Trp‐like proteins encoded in the *B. henselae* genome. The eight *trp*s located immediately downstream of the Brt family of RNAs show the greatest sequence homology and cluster together (red box). A neighbor joining tree was constructed for all 24 proteins using Jukes‐Cantor substitution. Bootstrap values are indicated in black font. Scale bar representing branch length is displayed below the tree

A closer examination of the genome loci for the nine *brt*s shows that all of them are immediately upstream of a coding region for a family of putative transcriptional regulatory proteins (*trp*s). There are two versions of the genome annotation of the Houston‐1 strain of *B. henselae* available online with some variation within the length of the coding region for these transcriptional regulators that results in slightly different size intergenic regions. It should be noted that the *trp8* gene downstream of *brt*8 appears to encode a pseudogene with a coding region still present, but disrupted by two stop codons. The other remaining eight *brt*s retain virtually the identical gene arrangement with respect to the downstream *trp* (Figure 4a). Sixteen additional Trp‐like proteins, with varying degrees of amino acid sequence and domain conservation compared to the Trps, are also found encoded throughout the *B. henselae* genome (Table S2). However, none of these gene copies are found adjacent to a Brt RNA like those identified with RNA‐seq. The number of *trp* genes found in various *Bartonella* species and whether they are linked to a *brt* sequence on the genome is shown in Table S3.

### 3.7 The Trps belong to a family of helix‐turn‐helix DNA‐binding proteins

The region located immediately downstream of each *brt* codes for a family of small DNA‐binding proteins (Trps). The deduced amino acid sequence from the Trp coding region downstream of each of the Brt RNAs (except the pseudogene behind *brt*8) is well conserved (Figure 4b). These proteins are small; varying in size from 108 to 152 amino acids and are annotated as xenobiotic response element‐like proteins (XREs), DNA‐binding proteins, transcriptional regulators, or hypothetical proteins (Alsmark et al., 2004). All have a XRE motif with a helix‐turn‐helix putative DNA‐binding domain located in the more conserved amino terminal half of the protein (Figure 4b blue box). XREs are a family of transcriptional regulators that have been shown to be involved in the biogenesis of type IV pili, flagella, and biofilm formation in other gram‐negative bacteria (Wang, Ye, Kumar, Gao, & Zhang, 2014). While eight of the nine Brt family members have a *trp* gene located downstream on the *B. henselae* chromosome (except Brt8 which is followed by a *trp* pseudogene), there are 16 additional *trp*‐like genes found scattered throughout the *B. henselae* genome (Table S2). It is interesting to note that phylogenetic analysis of the deduced amino acid sequence for all 24 putative Trps (or Trp‐like proteins) shows that all eight of the proteins that are encoded downstream of the Brt RNAs cluster together, branching out from a single node on the tree (Figure 4c). Locus BH02210 encodes a protein that represents the closest member that is not preceded upstream by a Brt RNA. The remaining 15 Trp‐like proteins are more distally related than those encoded by *trp1‐7* and *trp9*. They contain the conserved helix‐turn‐helix putative DNA‐binding domain but are poorly conserved throughout the remainder of the protein.

### 3.8 Overexpression of *trp*1 results in upregulation of *badA* and increased biofilm formation

**Figure 5:**
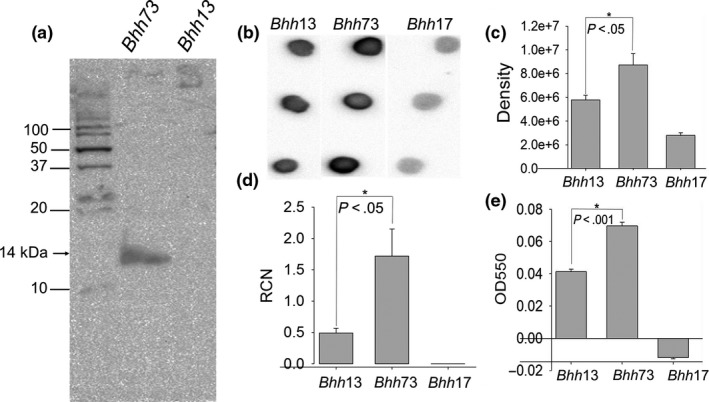
Expression of *badA*/BadA and biofilm formation in *Bhh*73 (overexpressing *trp1*–OE *trp*1, *Bh*/pNS2P_T__rc_ *trp1*). (a) Western blot with anti‐His tag antibody to detect Trp1, (b) Dot‐blot using rabbit anti‐BadA, (c). Densitometry of dot‐blot in panel (b and d) qRT‐PCR with *badA*‐specific primers showing relative copy number (RCN) compared to reference *rplD* mRNA, (e) biofilm formation. Strains are as described in Table 2 with *Bhh*17 (*ΔbadA*) and *Bhh13* (Houston‐1 pNS2P_*T*__*rc*_) as controls

Our RNA‐seq data showed that the Brt RNAs are highly transcribed, in contrast to the downstream Trp‐encoding genes, which are uniformly poorly transcribed (Figure 1a). This observation indicates transcriptional termination immediately upstream of the *trps*, at least under the conditions used for this study. Furthermore, the complex secondary structure noted at the 3′ end of the Brt1 RNA (Figure 3d) bears resemblance to those seen in riboswitches raising the possibility of a *cis*‐acting regulatory mechanism preventing transcription of the downstream transcriptional regulatory protein genes. Since the function of these Trp proteins is unknown in *B. henselae* and we hypothesize that they are potentially regulated by the Brt RNAs via a riboswitch mechanism, it is crucial to determine whether they would affect the expression of key virulence factors of *B. henselae*. To address this possibility, we cloned the downstream *trp1* gene into a plasmid with a strong constitutive promoter. We chose plasmid pNS2P_Trc_ since it contains the *trc* promoter which results in a high level of constitutive transcription in *B. henselae* since there is no lactose repressor gene (*lacI*) present in the genome (Gillaspie et al., 2009). For this reason, we cloned *trp1* into pNS2P_Trc_ to generate strain *Bhh*73 that constitutively overexpresses *trp1* (description in Table 2). *Bhh*73 was shown to express *trp1* efficiently with Trp1 easily detected by western blot using antibody to the 6XHis‐tag on the amino terminus of the fusion protein (Figure 5a). *Bhh73* was also noted to have a colony phenotype that is more adherent than the pNS2Trc vector control suggesting increased autoadherence due to elevated expression of *badA*. To test this hypothesis, *badA* expression in this strain was examined. *Bhh*73 had increased *badA* mRNA when compared to the control strain with the empty plasmid vector (Figure 5d). Furthermore, dot‐blot analysis using an anti‐BadA antibody showed increased BadA levels in the *trp1* overexpressing strain (Figure 5b,c). Finally, the *trp1* overexpressing strain had an increased ability to form biofilms (Figure 5e). Thus, *trp1* overexpression resulted in increased levels of *badA* mRNA and BadA protein and enhanced biofilm formation suggesting that Trp1 acts as a direct or indirect positive regulator of *badA*.

**Table 2:** *Bartonella henselae* strains and plasmids used for this study

Strain	Genotype/plasmid	Description/source
*Bh* Houston‐1	Wild‐type parental strain	From HIV+ patient (Regnery et al., 1992)
*Bhh*13	*Bh*/pNS2P_Trc_	Houston‐1/pNS2P_Trc_ control (Gillaspie et al., 2009)
*Bhh*17	*BhΔbadA*	Parental Houston‐1 strain with in‐frame deletion of *badA* (BH01510)(Lima et al., 2014)
*Bhh72*	*Bh*/pNS2P_Trc_asBrt1	Constitutive expression of antisense Brt1 RNA blocking Brt1 function
B*hh*73	*Bh*/pNS2P_Trc_ *trp1*	Constitutive expression of 6XHis‐tagged Trp1 (BH12870)

### 3.9 Brt1 knockdown results in upregulation of *badA* and increased biofilm formation

**Figure 6:**
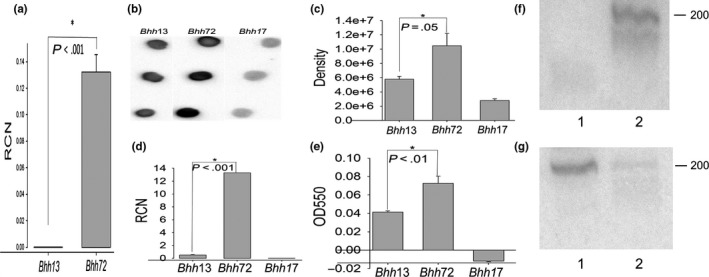
Expression of *badA*/BadA and biofilm formation in *Bhh*72 (antisense—ASBrt1, *Bh*/pNS2P_T__rc_asBrt1). (a) qRT‐PCR with primers specific for antisense Brt1 RNA showing relative copy number (RCN) compared to reference *rplD* mRNA, (b) Dot‐blot using rabbit anti‐BadA, (c). Densitometry of dot‐blot in panel (b and d) qRT‐PCR with *badA*‐specific primers, (e) biofilm formation. (f and g) Northern blots with *Bhh*13 RNA (lane 1) and *Bhh*72 RNA (lane 2). The probes used are specific to antisense Brt1 (asBrtProbe, panel f) and Brt RNA (BrtProbe, panel g). Strains are as described in Table 2 with *Bhh*17 (*ΔbadA*) and *Bhh13* (Houston‐1 pNS2P_*T*__*rc*_) as controls

Since high level expression of the Brt family of RNAs, their size, their complex secondary structure, and their location in noncoding regions of the *B. henselae* genome are suggestive of *trans*‐acting sRNA, we examined this possibility using an antisense (as) approach to block Brt function. Specifically, to determine if Brt1 functions as a small regulatory RNA that plays a role in gene regulation, we constructed an antisense (as) construct/strain designed to base‐pair and destabilize the Brt1 RNA. Since *trans*‐acting RNAs typically act by base‐pairing, we reasoned that asBrt1 RNA would block the function of Brt1 and possibly also destabilize the RNA and make it prone to degradation. Using primers that would only detect vector sequences found in the antisense Brt1 RNA, transcription of high levels of antisense Brt1 (Figure 6a) was noted in the strain of *Bh* harboring the asBrt1 plasmid (*Bhh*72, description in Table 2). A northern blot using probes specific for both antisense‐Brt1 RNA and Brt1 itself shows that *Bhh72* produces antisense Brt1 (Figure 6f, lane 2) but that Brt1 RNA appears to be degraded (Figure 6g, lane 2) when compared to the *Bhh*13 control with the empty vector (Figure 6g, lane 1). *Bhh*72 was also observed to have an adherent colony phenotype leading us to examine *badA* gene expression. *Bhh*72 had increased *badA* mRNA when compared to the control strain with the empty plasmid vector (Figure 6d). Furthermore, dot‐blot analysis using an anti‐BadA antibody showed increased BadA levels in the antisense strain (Figure 6b,c). Finally, the Brt1 antisense strain had an increased ability to form biofilms (Figure 6e). The absence of a longer Brt transcript in *Bhh*72 when using a Brt1 probe (Figure 6g, lane 2) suggests that the antisense RNA was not functioning by altering secondary structure of the Brt1 and allowing transcriptional read‐through into the downstream *trp1* gene.

To examine possible mRNA targets of Brt1, the RNApredator and IntaRNA prediction programs were run using Brt1 as the query and the genome of the Houston‐1 strain of *B. henselae* as the target (Busch et al., 2008; Eggenhofer et al., 2011). Multiple mRNA targets were predicted with the Brt1:mRNA duplex formation as stable as −11.1 kcal/mol for the *bepC* (BH13400) mRNA (Table 3). Additional *B. henselae* genes among the top predicted targets include a regulatory protein (BH11940) containing a conserved domain known to activate sigma factor 54 (Studholme & Dixon, 2003), the truncated copy of the *badA* gene (BH01490) as well as two copies of the filamentous hemagglutinin genes (BH06670 and BH07150) and four ABC transporter genes (BH00170, BH01900, BH12230, and BH15040). In addition, genes encoding VirB type IV secretion system components VirB6 (BH13300) and VirB9 (BH13330) were predicted targets of Brt1 (Table 3). Three histidine kinases including BatS (BH00610), an orphan histidine kinase/response regulator (BH09640) and the general stress response kinase (BH13820) were also among the predicted targets for Brt1 (Table 3). The full‐length copy of the *badA* gene (BH01510) mRNA was predicted to interact with Brt1 but with a predicted ∆G of −1.73 kcal/mol indicating low stability of this interaction. The top 100 predicted mRNA targets from each algorithm are shown in Tables S4 and S5.

**Table 3:** Selected predicted targets of Brt1 RNA^a^

Gene locus	Gene name/annotation	Function	Algorithm	∆G kcal/mol
BH13400	BepC	Pathogenesis, T4SS effector	IntaRNA	−11.1
BH01900	ABC transporter	Ferric uptake	IntaRNA	−10.0
BH16450	PhaC antiporter	pH adaptation, H+/K+ efflux	IntaRNA	−9.7^b^
BH13330	VirB9	Pathogenesis, T4SS	IntaRNA	−9.7
BH05790	LipA	Lipoic acid metabolism	IntaRNA	−9.7^b^
BH13820	Histidine kinase	General stress response	IntaRNA	−9.6
BH00170	ABC transporter	LPS biosynthesis/export	IntaRNA	−8.9
BH00530	HemN	Heme biosynthesis	IntaRNA	−8.8
BH09640	Histidine kinase	Two‐component system	IntaRNA	−8.6
BH13300	VirB6	Pathogenesis, T4SS	IntaRNA	−8.1
BH06280	Omp89	LPS biosynthesis, adhesin	IntaRNA	−7.9
BH01980	Fur2	Iron response regulator	IntaRNA	−7.9
BH11940	Regulatory protein	ATPase	RNAPredator	−4.0
BH12230	ABC transporter	Transmembrane transport	RNAPredator	−3.7
BH01490	BadA1	Adhesin, truncated BadA	RNAPredator	−3.3
BH15040	ABC transporter	LPS metabolism	RNAPredator	−3.0
BH07150	FhaB4	Filamentous hemagglutinin	RNAPredator	−2.9^b^
BH06670	FhaB2	Filamentous hemagglutinin	RNAPredator	−2.9
BH04040	NhaA	pH regulation	RNAPredator	−2.9
BH00610	BatS	Histidine kinase	RNAPredator	−2.6

Genome loci refer to *B. henselae* Houston‐1 strain genome sequence accession number BX897699.

a A complete list of the top predicted targets may be found in supportive information Table S4.

b Predicted by both IntaRNA and RNAPredator algorithms.

## 4 Discussion

The genus *Bartonella* consists of over 30 species of bacteria found in a wide range of animal hosts with most thought to be transmitted by an arthropod vector (Buffet, Kosoy, & Vayssier‐Taussat, 2013). *B. henselae*,* B. quintana*, and *B. bacilliformis* are the species most commonly known to cause human disease. While *B. quintana* and *B. bacilliformis* use humans as their sole natural reservoir, *B. henselae* is found in cats, and incidental transmission of this bacterium to humans results in disease (Anderson & Neuman, 1997). Thus, *Bartonella* species must be able to quickly adapt to the drastically different conditions associated with transmission from a vertebrate animal to an arthropod vector and back into another host. Several regulatory proteins of *Bartonella* have been proposed to play a role in this response (Battisti et al., 2006; Quebatte et al., 2010, 2013; Roden et al., 2012). The adaptive response is also thought to be controlled by an alternate sigma factor RpoE of the general stress response system found in both *B. quintana* (Abromaitis & Koehler, 2013; Abromaitis et al., 2013) and *B. henselae* (Tu et al., 2016). Genes in the *B. henselae* general stress response regulon include the hemin‐binding proteins and the *badA* adhesin (Tu et al., 2016). In addition, to this general stress response system, two‐component regulatory systems have also been described in *Bartonella* species. The BatR/S two‐component system has been shown to control the VirB type IV secretion system of *B. henselae* and the associated *Bartonella* effector proteins as well as playing an undefined role in controlling *badA* expression (Omasits et al., 2013; Quebatte et al., 2010, 2013). Despite these elegant descriptions of gene regulation in *B. henselae* by two‐component systems and an alternate sigma factor, the role of RNAs in gene regulation by this bacterium remains unreported.

Here, we describe a family of nine unannotated and highly transcribed RNAs designated as Brt RNAs using RNA‐seq. The Brt1 member of this family exhibits properties of sRNAs such as the high levels of transcription, small size, and highly stable predicted secondary structure. A search of the NCBI genome database revealed that this RNA family is specific to only *Bartonella* species, although not every species harbors Brt RNAs as they were not found in *B. bacilliformis* or *B. rochalimae*. In addition, the RNA gene copy number varies between species; the human‐specific species either harbor none (*B. bacilliformis*) or two (*B. quintana*), while other species contain a wide range from a moderate number (*B. henselae*) to the highest number of copies/genome in the rodent‐associated *B. tribocorum* (Table 1). The copy number generally correlates well with genome size and may be indicative of gene duplication by transposition or horizontal gene transfer. This is further supported by the presence of the Brt‐coding sequences in only a small part of the *B. henselae* genome that has been described as highly plastic and that very likely contains several horizontally acquired mobile genetic elements (Lindroos et al., 2006).

Interestingly, all nine Brt RNAs are found upstream of genes annotated as XRE transcriptional regulators that we designated as *trps* in *B. henselae*. The fact that this gene arrangement is highly conserved suggests that both the Brt RNA gene and the *trp* gene may have been acquired and/or duplicated together. The intergenic region between each of the nine Brts and its cognate *trp* is very short ranging from 10 to 18 nucleotides. This operon‐like gene arrangement, wherein the first gene is an RNA with no promoter region located before the second gene, suggests a common or paired function of these two gene products. It is possible that Brt RNAs are merely a 5′ untranslated RNA region for the downstream associated *trp*. However, in each of the pairs of Brts/*trps*, very little *trp* RNA was observed by RNA‐seq despite much higher levels of transcription of the upstream Brt (Figure 1). The stem‐and‐loop structures at the 3′ end of the Brt RNA transcript and the genomic organization of the Brt RNA relative to the downstream *trp* genes are reminiscent of a riboswitch mechanism in which transcription termination of the downstream *trp* gene is dependent on the secondary structure conformation of the Brt RNA. However, transcriptional read‐through from Brt into *trp* did not happen to any significant degree under the growth conditions used for bacterial culture for our RNA‐seq studies. We are hypothesizing that under yet undefined conditions, transcriptional read‐through into *trp* occurs possibly upon binding of a ligand allowing the Brt riboswitch to change conformation to an antitermination structure allowing read‐through transcription of the *trp* gene.

To examine the effect of expressing the otherwise silent *trp1* gene, we expressed *trp1* from a strong promoter in a plasmid producing Trp1 as a 6X His‐tagged fusion protein. The resulting overexpressing strain produced more *badA* mRNA and BadA protein. Additionally, the *trp1* overexpressing strain was more efficient at biofilm formation than the control strain. Thus, Trp1 appears to be a positive regulator of *badA*. The deduced amino acid sequences of the Trp family of proteins indicates a variable level of sequence identity (34–100%), with the amino terminal to central regions of the protein, the most highly conserved. All of the Trps have a helix‐turn‐helix DNA‐binding domain located in this conserved region and most are annotated in the *B. henselae* genome sequence as xenobiotic response elements (XREs). Bacterial XREs are DNA‐binding proteins that have a conserved positively charged helix that is thought to bind the major groove of DNA (Wang et al., 2014). It is interesting to note that XREs in *Pseudomonas aeruginosa* have been shown to regulate genes controlling the biogenesis of type IV pili, flagella, and biofilms (Wang et al., 2014). Furthermore, in *P. aeruginosa*, the XRE acts through a small RNA molecule *rsmZ* to modulate motility and biofilm formation. A transcriptional regulator annotated as an XRE with a helix‐turn‐helix domain is required for conjugative transfer in the alpha‐proteobacterium *Rhizobium etli* (Lopez‐Fuentes, Torres‐Tejerizo, Cervantes, & Brom, 2014). In addition, to the eight *trp* genes located immediately downstream of their cognate Brt RNA, there are 16 additional *trp*‐like genes located in the *B. henselae* genome (Table S2) that are more distally related (Figure 4). The function of this large Trp protein family in *Bartonella* remains to be elucidated.

The high levels of Brt RNA transcription combined with their size and secondary structure are also suggestive of trans‐acting sRNAs. To test this hypothesis, we attempted to make a Brt1 deletion strain of *B. henselae*. We selected Brt1 since this RNA is transcribed at a much higher level that the other eight Brt RNAs (Figure 3a) and gene deletion of Brt1 would likely have a more pronounced effect on the phenotype than deletion of any of the other Brt genes. Despite three attempts, we were not successful in obtaining a Brt1 deletion mutant raising the possibility that Brt1 may be an essential RNA gene. We then turned to an antisense approach to determine if Brt1 plays a role in gene regulation, by constructing an antisense (as) construct/strain designed to base‐pair and destabilize the Brt1 RNA. Since trans‐acting RNAs typically act by base‐pairing, we reasoned that asBrt1 RNA would block the function of Brt1 and possibly destabilize the RNA, thus making it prone to degradation. Our results indicate that this antisense‐based approach did result in diminished Brt1 RNA, suggesting that base‐pairing targeted this RNA for degradation. The strain of *B. henselae* harboring the asBrt1 plasmid (*Bhh*72) was noticed to have an adherent colony phenotype leading us to examine *badA* gene expression. *Bhh*72 had increased *badA* mRNA and BadA protein as determined by dot‐blot analysis and was further shown to have an increased ability to form biofilms. It is possible that this increased *badA* expression is due to the asBrt1 functioning to alter secondary structure of the Brt1 and allowing transcriptional read‐through into the downstream *trp1* gene. Further studies to determine the sequence and abundance of Brt and *trp1* RNA species are needed to answer this question and test our hypothesis that Brt1 functions in trans to negatively regulate *badA* in a manner that is independent of *trp1* transcription.

**Figure 7:**
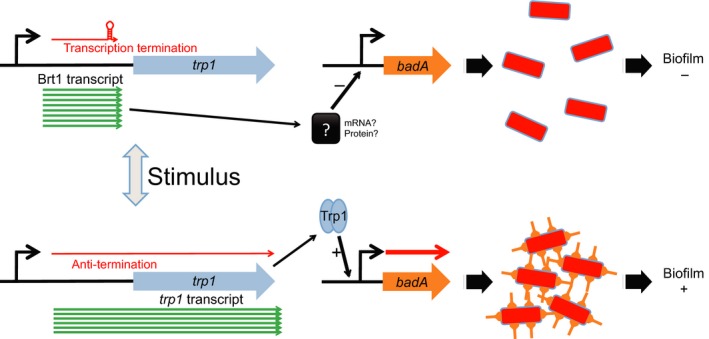
Proposed model of Brt1/Trp1 regulation. Under laboratory growth conditions, Brt1 is highly transcribed and functions as a *trans*‐acting RNA possibly to limit *badA* expression, autoaggregation, and biofilm formation (top). A stimulus activates secondary structure changes in the 3′ end of the Brt1 RNA resulting in transcription of the downstream *trp1*. Trp1 serves as a DNA‐binding protein that activates *badA* gene expression facilitating autoaggregation and biofilm formation in the absence of functional Brt1 (bottom)

We have data supporting the possible function of Brt1 both as a trans‐acting sRNA that negatively controls *badA* expression and as a possible riboswitch controlling transcription of the downstream *trp1*. While we have not yet identified ligands or conditions that activate this putative riboswitch, it is clear that *trp*1 expressed on a plasmid results in upregulation of *badA*, the same gene negatively regulated by the associated Brt1 RNA. Based on the experimental data presented in this study, we propose a model for regulation of *badA* by Brt1/Trp1. Firstly, Brt1 functions as a *trans*‐acting sRNA to repress *badA* expression possibly targeting the *badA* mRNA directly or through an intermediate RNA. Secondly, we hypothesize that Brt1 also acts on *badA* by a second mechanism in which a *cis*‐acting riboswitch on the 3′ end of Brt1 adopts an antitermination configuration to allow transcription of the downstream *trp1*. Trp1 in turn functions as a DNA‐binding protein to activate *badA* expression which facilitates autoaggregation and biofilm formation (Figure 7). Whether Trp1 regulates *badA* directly or through an intermediate remains to be determined at this point. Thus, under laboratory growth conditions, Brt1 is abundantly transcribed and we get a basal level of *badA* transcription that can be further upregulated by the activation of the *trp1* gene. In this model, activation of an antitermination switch at the 3′ end of Brt1 results in a full‐length transcript that includes *trp1* allowing expression of high levels of Trp1 and reducing or eliminating the abundant short Brt1 RNA that represses *badA* expression. This dual‐function model of regulation by the Brt1/Trp1 system is novel and includes both an RNA‐based negative regulatory system that is transcriptionally coupled to the expression of the Trp1 DNA‐binding protein that regulates *badA* in a positive manner. To the best of our knowledge, a similar dual‐function RNA (riboswitch RNA that is also a trans‐acting regulatory RNA) has only been described for the SreA and SreB trans‐acting riboswitches that controls virulence in *Listeria monocytogenes* (Loh et al., 2009).

Questions remain to be answered about how the Brt1 RNA and the Trp1 protein mediate the effect on the *badA* gene. Since Brt1 is a small RNA, we hypothesize that it binds a target, perhaps *badA* mRNA directly or perhaps another target gene that then affects *badA* expression. We are in the process of examining Brt1 RNA interaction with other RNAs in *B. henselae*. When the Brt1 RNA was used as a query sequence to predict mRNA targets in *B. henselae* using the RNApredator and IntaRNA algorithms, several genes were found including the truncated version of *badA* (BH01490, Table 3). However, the full‐length *badA* gene (BH01510) was not among the top targets and was predicted to only weakly interact with Brt1, raising the possibility that Brt1 acts on *badA* by an indirect mechanism. Alternatively, it is also possible that an RNA‐binding protein, such as Hfq, is involved in these interactions and allows Brt1 to bind a target mRNA in the absence of extensive base‐pairing. It should also be noted that discrepancies were observed between these two sRNA target prediction algorithms and while some targets were in common (PhaC pH adaptation transporter and LipA lipoyl synthase), most targets were unique to one of the algorithms. Specific target mRNAs of Brt1 and the other Brt family RNAs will require experimental documentation. Similarly, the function of Trp1 could be direct or indirect. We do not yet know if Trp1 functions by binding upstream of *badA* or another target gene that affects *badA* gene expression. We have previously shown that the general stress response system of *B. henselae* controls *badA* gene expression and it is possible that Brt1 and/or Trp1 acts through this regulatory system.

The gene expression patterns generated by RNA‐seq, qRT‐PCR, and dot‐blots described in this report use *B. henselae* grown for 72 hr at 37°C in the presence of 5% CO_2_ in standard culture media. It is certainly possible that under different growth conditions, the expression patterns of the Brt RNA, the Trp protein genes, and the *badA* gene might be different. We hypothesize that different growth conditions, perhaps mimicking the cat flea vector, would result in differential expression patterns. These conditions would be predicted to result in diminished *badA* expression since *badA* is known to play a critical role in interacting with vertebrate cells, but its role for growth in the cat flea vector has not been established and increased adherence may be unnecessary. Alternatively, after extended growth under the conditions of the cat flea vector, the bacteria might be primed for infection of the vertebrate host by upregulating genes such as *badA*.

The multiple copies of Brt RNA and Trp protein genes in the *B. henselae* genome are intriguing. The Brt RNAs are found in all *Bartonella* species except *B. bacilliformis* and *B. rochalimae;* however, in most species, there are multiple copies of these RNAs suggesting that their function is important to the overall fitness and survival of these bacteria. It remains to be seen if the multiple copies of the Brt RNA function in a redundant or complementary manner. The multiple copies of the Brt family of RNAs also presents a problem in dissecting individual Brt and Trp function, since construction of deletion mutants in *B. henselae* is challenging. We are still attempting to make a ∆Brt1 strain to examine the phenotype and *badA* expression in the absence of this RNA. It is possible that increased compensatory expression of one or more of the other Brt family RNA will be observed in such a mutant. The presence of the eight *trp* genes following their cognate Brt RNA as well as 16 other *trp*‐like genes makes dissecting *trp* function and even more daunting task. Thus, deleting all Brts and their associated *trps* is impractical.

Since *B. henselae* has been described to autoagglutinate in a manner that is dependent upon expression of *badA* (Riess et al., 2007), we elected to extend this study to include the formation of biofilms. BadA is found in the genomes of all characterized *Bartonella* species (O'Rourke, Schmidgen, Kaiser, Linke, & Kempf, 2011). While it was not the specific focus of this study, this is the first report describing the requirement of BadA in biofilm formation by *B. henselae*. Our *B. henselae* Houston‐1 *badA* deletion mutant (*Bhh*17) consistently demonstrated an impaired ability to form biofilms in our assay and served as a negative control for the biofilm assay. Considering the well‐characterized role of BadA in autoagglutination, it is reasonable to expect that the surface location and adherent nature of BadA results in a role for this protein in biofilm formation. The requirement for BadA to form biofilms makes it tempting to speculate on the role of this protein in establishing the vegetative masses described in patients with infective endocarditis caused by *B. henselae* (see (Edouard et al., 2015) for review). BadA belongs to the TAA‐family of proteins that are found in many other gram‐negative bacteria including *Escherichia coli*,* Yersinia enterocolitica*,* Haemophilus influenzae*, and *Neisseria meningitidis* (see (Linke, Riess, Autenrieth, Lupas, & Kempf, 2006) for review).

The long observed phenomenon of autoadherence and the sticky and adherent colony phenotypes reported in fresh clinical isolates of *B. henselae* (and very likely other *Bartonella* species) is thought to be due to the variable expression of *badA* (Riess et al., 2007). The Brt/Trp regulatory system described in this report may explain the change in colony phenotype upon repeated culture of *B. henselae* in the laboratory. It would be interesting to examine fresh isolates of *B. henselae* with the same strain that has been repeatedly passaged to examine not only *badA* expression but also *brt*1 and *trp1* transcript levels. Recently, the presence of BadA in *B. henselae* strains was shown to adversely affect the expression of the VirB type IV secretion system genes and the function of the cognate effectors (Lu et al., 2013). In the presence of full‐length BadA protein, VirB expression was reduced and it could be restored upon deletion of *badA* (Lu et al., 2013). Thus, the presence of these two pathogenicity factors appears to be coordinated. It would be interesting to examine the role of Brt RNAs and Trp proteins in coordinating the expression of *badA* and the genes encoding the VirB type IV secretion system. Interestingly, some of the VirB type IV secretion system components including *bepC*,* virB6*, and *virB9* were predicted targets of Brt1. Of note, the *B. bacilliformis* genome does not contain the Brt RNA genes and also lacks the VirB type IV secretion system. Thus, Brt RNAs are absent in one of the species of *Bartonella* that does not have a need to coordinately regulate these two systems.

## Conflict of Interest

The authors declare no potential conflict of interest.
