# Supplementary material for: A family of genus‐specific RNAs in tandem with DNA‐binding proteins control expression of the badA major virulence factor gene in Bartonella henselae
Source: Microbiologyopen. 2016 Oct 28;6(2):e00420. doi: 10.1002/mbo3.420 (PMC5387305; doi:10.1002/mbo3.420)
Supplement: Supplementary file 1 [file MBO3-6-na-s001.docx]

**Supporting Information**

**Table S1. Size and location of Brt RNA genes in the *B. henselae* genome sequence**

| Brt | Size (nucleotides) | Start | End |
| --- | --- | --- | --- |
| 1 | 195 | 1439057 | 1439251 |
| 2 | 203 | 1552533* | 1552331 |
| 3 | 200 | 1559332* | 1559133 |
| 4 | 200 | 1626162 | 1626361 |
| 5 | 200 | 1632032 | 1632231 |
| 6 | 195 | 1642011 | 1642205 |
| 7 | 195 | 1642823 | 1643017 |
| 8 | 202 | 1643670 | 1643871 |
| 9 | 200 | 1715820 | 1716019 |

*Brt2 and Brt3 are transcribed in the opposite strand in the reverse orientation (see Fig. 1). Genome numbering refers to *B. henselae* Houston-1 strain genome sequence accession number BX897699.

**
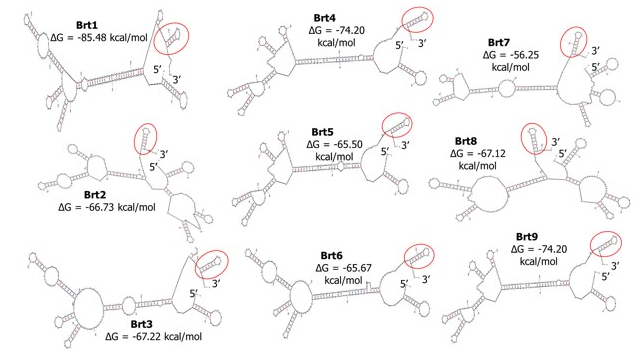
**

**Figure S1. Secondary Structure predictions for the nine Brt family RNAs.** The calculated Gibbs free energy (∆G) is indicated. The putative terminator/riboswitch is indicated by the red oval.

**File S1. Identification of Brt RNAs**

RNAseq was performed on *B. henselae*, grown in Schneider’s liquid medium at 37ºC with 5% CO_2_ for 72 hours. One library containing a total of 2,537,304 reads was generated, of which 2,223,598 (88%) represented ribosomal RNA. The rRNA reads were removed and the remaining 313,706 reads were mapped to the *B. henselae* Houston-1 genome (NC_005956) using CLC Genomics Workbench default alignment parameters (mismatch cost = 2, insertion/deletion cost = 3, length fraction =0.5, similarity fraction = 0.8). While the depth of coverage across the genome was low, transcripts from highly expressed genes were visible. Through a visual inspection of the genome we identified a number of highly expressed transcripts that did not map to annotated genes. The majority of these transcripts were located in a novel region of the genome previously identified by Omastis *et al.* (Genome Res. 2013 23:1916-27) as having a number of highly expressed genes for which no protein product could be detected. Of particular interest we noted that two of these highly expressed transcripts had a 100% identical sequence despite being located approx. 89 kilobase pairs apart on the genome (these RNAs were eventually named Brt4 and Brt9). To investigate if additional homologues were encoded on the genome we performed a BLAST search (using the Brt4/9 sequence) and identified 7 additional homologues with DNA sequence identity greater than 79%. The 9 homologous sequences were all highly expressed in the RNAseq data set and were named Brt1-9 in the order in which they appear on the genome.

To quantify expression of the Brt RNAs using the RNAseq data set, new annotations were added to the *B. henselae* Houston-1 genome for each of the 9 Brt genes, as previously described in Carroll *et al.* (MBio. 2016 7:e01990-15). RNAseq data analysis was then carried out to generate expression values (in RPKM) for each gene in the genome. One Brt gene (Brt1) was amongst the top 10 most highly expressed genes in the genome (see table below). The lowest expressed Brt transcript (Brt7) generated an RPKM value of 707. Although almost two orders of magnitude lower than that of Brt1, the expression of Brt7 was in the 90^th^ percentile of highly expressed genes in *B. henselae.*

| Name | Expression value (RPKM) |
| --- | --- |
| BH11030 | 778,710 |
| BH08960 | 601,117 |
| rnpB | 218,587 |
| Brt1 | **65,949** |
| hbpA | 24,867 |
| BH00970 | 24,473 |
| BH01420 | 22,149 |
| BH10670 | 16,665 |
| BH06030 | 16,493 |
| BH13770 | 15,641 |

**Table S2. Genome locus of the Trp and Trp-like proteins in the *B. henselae* genome.***

| **Locus** | **Designation** | **Size (aa)** | **Genome Annotation** |
| --- | --- | --- | --- |
| BH01460 | Trp-like | 167 | Hypothetical protein |
| BH01480 | Trp-like | 180 | Hypothetical protein |
| BH02210 | Trp-like | 138 | Transcriptional regulator |
| BH03120 | Trp-like | 109 | Virulence associated protein |
| BH03380 | Trp-like | 98 | Virulence associated protein A |
| BH03560 | Trp-like | 96 | Virulence associated protein A |
| BH03650 | Trp-like | 205 | Phage repressor protein |
| BH06620 | Trp-like | 102 | Virulence associated protein |
| BH06820 | Trp-like | 99 | Virulence associated protein A |
| BH06930 | Trp-like | 112 | Hypothetical protein |
| BH07730 | Trp-like | 82 | Hypothetical protein |
| BH09200 | Trp-like | 102 | Hypothetical protein |
| BH09490 | Trp-like | 96 | Virulence associated protein A |
| BH11420 | Trp-like | 179 | Hypothetical protein |
| BH11470 | Trp-like | 104 | Hypothetical protein |
| BH12870 | Trp1 | 118 | DNA-binding protein |
| BH13560 | Trp2 | 119 | Transcriptional regulator |
| BH13590 | Trp3 | 125 | Transcriptional regulator |
| BH14160 | Trp4 | 128 | Transcriptional regulator |
| BH14230 | Trp5 | 108 | Transcriptional regulator |
| BH14270 | Trp-like | 75 | Virulence associated protein |
| BH14370 | Trp6 | 152 | DNA-binding protein |
| BH14380 | Trp7 | 152 | DNA-binding protein |
| BH14970 | Trp9 | 130 | Transcriptional regulator |

*****Numbering convention is such that Trp1 follows Brt1 RNA, Trp2 follows Brt2 RNA, etc. Proteins designated as Trp-like do not follow a Brt family RNA. *B. henselae* Houston-1 strain genome sequence accession number BX897699.

**Table S3. Number of Brt and *trp* gene copies in the genomes of various *Bartonella* species**

| **Strain** | **# of Brt RNAs** | **# of Trps** | **# of Trps associated**  **with Brt** | **Orphan Trps** |
| --- | --- | --- | --- | --- |
| *Bartonella bacilliformis* | 0 | 1 | 0 | 1 |
| *Bartonella rochalimae* ATCC BAA-1498 | 0 | 1 | 0 | 1 |
| *Bartonella quintana* str. Toulouse | 2 | 1 | 0 | 1 |
| *Bartonella quintana* str. RM-11 | 2 | 1 | 0 | 1 |
| *Bartonella schoenbuchensis* str. MVT06 | 2 | 2 | 2 | 0 |
| *Bartonella schoenbuchensis* R1 | 3 | 6 | 4 | 2 |
| *Bartonella henselae* BM1374165 | 7 | 8 | 8 | 0 |
| *Bartonella henselae* BM1374163 | 7 | 8 | 7 | 1 |
| *Bartonella henselae* str. Houston-1 | 9 | 24 | 8 | 16 |
| *Bartonella vinsonii*  *subsp. Berkhoffii* str. Winnie | 8 | 3 | 3 | 0 |
| *Bartonella grahamii* as4aup | 26 + 1  plasmid | 17 | 16 | 1 |
| *Bartonella tribocorum* str. BM1374166 | 33 | 24 | 22 | 2 |
| *Bartonella tribocorum* CIP 105476 | 34 **RNAs** | 21 | 20 | 1 |

To determine if the HTH-XRE conserved domain exist in other *Bartonella* species, this conserved region from each Trp found in *B. henselae* were identified using NCBI’s conserved domain feature. The resulting conserved domain (e.g. residues 14-71 for Trp1) was used as the query sequence. The NCBI Basic Local Alignment Search Tool (BLAST) was used to compare the query sequence against the available genomes of individual *Bartonella* species and sequences which have at least a 50% similarity to the query sequence and bear the conserved HTH-XRE domain were identified. To differentiate between a Brt located immediately upstream of a linked *trp* gene in the genome and an orphan *trp* not associated with a Brt, the region upstream of each *trp* was searched for the presence or absence of sequences with homology to each Brt from *B. henselae*.

**Table S4. mRNA targets for Brt1 predicted with IntaRNA Algorithm**

| [p-value](http://rna.informatik.uni-freiburg.de/IntaRNA/Result.jsp?sortBy=p_value&toolName=IntaRNA&jobID=7423155&numRows=all) | [Target](http://rna.informatik.uni-freiburg.de/IntaRNA/Result.jsp?sortBy=target_name&toolName=IntaRNA&jobID=7423155&numRows=all) | [Position](http://rna.informatik.uni-freiburg.de/IntaRNA/Result.jsp?sortBy=target_position&toolName=IntaRNA&jobID=7423155&numRows=all) | [Energy](http://rna.informatik.uni-freiburg.de/IntaRNA/Result.jsp?sortBy=energy&toolName=IntaRNA&jobID=7423155&numRows=all) | [Gene](http://rna.informatik.uni-freiburg.de/IntaRNA/Result.jsp?sortBy=gene_name&toolName=IntaRNA&jobID=7423155&numRows=all) | [Annotation](http://rna.informatik.uni-freiburg.de/IntaRNA/Result.jsp?sortBy=annotation&toolName=IntaRNA&jobID=7423155&numRows=all) |
| --- | --- | --- | --- | --- | --- |
| 0.0024916 | [BH08540](http://www.ncbi.nlm.nih.gov/gene/?term=2864850) | 118 -- 137 | -12.0401 | ubiH | 2-octaprenyl-6-methoxyphenyl hydroxylase |
| 0.0034814 | [BH16070](http://www.ncbi.nlm.nih.gov/gene/?term=2865090) | 105 -- 135 | -11.5638 |  |  |
| 0.0046833 | [BH13400](http://www.ncbi.nlm.nih.gov/gene/?term=2865508) | 140 -- 149 | -11.1359 |  | cell filamentation protein |
| 0.0059743 | [BH03950](http://www.ncbi.nlm.nih.gov/gene/?term=2865658) | 139 -- 149 | -10.7806 |  |  |
| 0.0060637 | [BH08360](http://www.ncbi.nlm.nih.gov/gene/?term=2865184) | 100 -- 109 | -10.7588 | sfsA | sugar fermentation stimulation protein A |
| 0.0065873 | [BH06790](http://www.ncbi.nlm.nih.gov/gene/?term=2864643) | 30 -- 50 | -10.637 |  |  |
| 0.0068025 | [BH04320](http://www.ncbi.nlm.nih.gov/gene/?term=2866079) | 134 -- 149 | -10.5896 | aroC | chorismate synthase |
| 0.0069173 | [BH03890](http://www.ncbi.nlm.nih.gov/gene/?term=2864742) | 43 -- 72 | -10.5649 | glnA1 | glutamine synthetase |
| 0.0070102 | [BH13490](http://www.ncbi.nlm.nih.gov/gene/?term=2865515) | 43 -- 50 | -10.5452 |  |  |
| 0.0072524 | [BH03530](http://www.ncbi.nlm.nih.gov/gene/?term=2865923) | 134 -- 147 | -10.495 |  |  |
| 0.008108 | [BH00490](http://www.ncbi.nlm.nih.gov/gene/?term=2865561) | 121 -- 135 | -10.3296 | infC | translation initiation factor IF-3 |
| 0.0091553 | [BH08850](http://www.ncbi.nlm.nih.gov/gene/?term=2864984) | 59 -- 70 | -10.1485 | nuoK | NADH dehydrogenase subunit K |
| 0.0094634 | [BH04330](http://www.ncbi.nlm.nih.gov/gene/?term=2864799) | 6 -- 24 | -10.099 | ribA | GTP cyclohydrolase II |
| 0.0104329 | [BH01900](http://www.ncbi.nlm.nih.gov/gene/?term=2864615) | 119 -- 145 | -9.95267 |  | ABC transporter permease |
| 0.0121758 | [BH16450](http://www.ncbi.nlm.nih.gov/gene/?term=2865025) | 1 -- 12 | -9.71956 | phaC | monovalent cation/H+ antiporter subunit C |
| 0.0124866 | [BH13330](http://www.ncbi.nlm.nih.gov/gene/?term=2865830) | 122 -- 135 | -9.68137 | virB9 | virB9 protein |
| 0.0126224 | [BH05790](http://www.ncbi.nlm.nih.gov/gene/?term=2865584) | 43 -- 52 | -9.66496 | lipA | lipoyl synthase |
| 0.0130181 | [BH15800](http://www.ncbi.nlm.nih.gov/gene/?term=2865892) | 101 -- 130 | -9.61811 | sdhC | succinate dehydrogenase cytochrome b560 subunit |
| 0.0135576 | [BH13820](http://www.ncbi.nlm.nih.gov/gene/?term=2864879) | 52 -- 61 | -9.55636 |  | GSR HK |
| 0.0170016 | [BH06990](http://www.ncbi.nlm.nih.gov/gene/?term=2864923) | 137 -- 148 | -9.20998 |  |  |
| 0.0181576 | [BH09180](http://www.ncbi.nlm.nih.gov/gene/?term=2864467) | 118 -- 128 | -9.10861 |  |  |
| 0.0182383 | [BH03210](http://www.ncbi.nlm.nih.gov/gene/?term=2865645) | 9 -- 29 | -9.10176 |  |  |
| 0.0184184 | [BH00880](http://www.ncbi.nlm.nih.gov/gene/?term=2864811) | 129 -- 138 | -9.08659 | yfeC | Iron transport system membrane protein yfeC |
| 0.0198133 | [BH09730](http://www.ncbi.nlm.nih.gov/gene/?term=2864777) | 7 -- 24 | -8.97361 | purQ | phosphoribosylformylglycinamidine synthase I |
| 0.020296 | [BH10960](http://www.ncbi.nlm.nih.gov/gene/?term=2865352) | 104 -- 116 | -8.93627 | hflK | protease subunit hflK |
| 0.0206781 | [BH00170](http://www.ncbi.nlm.nih.gov/gene/?term=2865955) | 109 -- 115 | -8.90731 |  | ABC transporter/ATP-binding protein |
| 0.0225771 | [BH00530](http://www.ncbi.nlm.nih.gov/gene/?term=2865349) | 30 -- 43 | -8.77049 | hemN | coproporphyrinogen III oxidase |
| 0.0226183 | [BH07060](http://www.ncbi.nlm.nih.gov/gene/?term=2865016) | 59 -- 75 | -8.76764 | pemK | PemK protein |
| 0.0239609 | [BH07180](http://www.ncbi.nlm.nih.gov/gene/?term=2864936) | 30 -- 50 | -8.6775 |  |  |
| 0.0245717 | [BH06330](http://www.ncbi.nlm.nih.gov/gene/?term=2865599) | 121 -- 127 | -8.63806 | lpxB | lipid-A-disaccharide synthase |
| 0.0255017 | [BH02430](http://www.ncbi.nlm.nih.gov/gene/?term=2865252) | 97 -- 116 | -8.57976 |  | phosphate binding protein |
| 0.0256071 | [BH09640](http://www.ncbi.nlm.nih.gov/gene/?term=2864759) | 139 -- 150 | -8.57328 |  | sensor histidine kinase |
| 0.0256096 | [BH10110](http://www.ncbi.nlm.nih.gov/gene/?term=2865570) | 12 -- 37 | -8.57313 | coaD | phosphopantetheine adenylyltransferase |
| 0.0273404 | [BH12350](http://www.ncbi.nlm.nih.gov/gene/?term=2866116) | 97 -- 103 | -8.47019 |  |  |
| 0.0284625 | [BH09540](http://www.ncbi.nlm.nih.gov/gene/?term=2864500) | 43 -- 58 | -8.4067 |  |  |
| 0.0286954 | [BH16220](http://www.ncbi.nlm.nih.gov/gene/?term=2864663) | 116 -- 137 | -8.39382 | holA | DNA polymerase III subunit delta |
| 0.0287112 | [BH15230](http://www.ncbi.nlm.nih.gov/gene/?term=2865170) | 137 -- 150 | -8.39295 | fdxA | ferredoxin II |
| 0.0297637 | [BH03510](http://www.ncbi.nlm.nih.gov/gene/?term=2865921) | 122 -- 130 | -8.33597 |  | DNA-binding protein |
| 0.0298107 | [BH09220](http://www.ncbi.nlm.nih.gov/gene/?term=2864471) | 105 -- 135 | -8.33347 |  |  |
| 0.0329846 | [BH01690](http://www.ncbi.nlm.nih.gov/gene/?term=2865518) | 55 -- 62 | -8.17272 | ppa | inorganic pyrophosphatase |
| 0.0330714 | [BH03280](http://www.ncbi.nlm.nih.gov/gene/?term=2865652) | 32 -- 38 | -8.16853 |  |  |
| 0.0351854 | [BH13300](http://www.ncbi.nlm.nih.gov/gene/?term=2865828) | 99 -- 121 | -8.0696 | virB6 | virB protein |
| 0.0369858 | [BH00780](http://www.ncbi.nlm.nih.gov/gene/?term=2864680) | 103 -- 112 | -7.98966 |  |  |
| 0.0372881 | [BH12330](http://www.ncbi.nlm.nih.gov/gene/?term=2865532) | 36 -- 50 | -7.9766 | proC | pyrroline-5-carboxylate reductase |
| 0.0374639 | [BH06780](http://www.ncbi.nlm.nih.gov/gene/?term=2864642) | 24 -- 40 | -7.96905 |  |  |
| 0.0380667 | [BH04700](http://www.ncbi.nlm.nih.gov/gene/?term=2865694) | 38 -- 45 | -7.94341 |  |  |
| 0.039614 | [BH06280](http://www.ncbi.nlm.nih.gov/gene/?term=2864991) | 102 -- 126 | -7.8793 | omp89 |  |
| 0.0403987 | [BH01980](http://www.ncbi.nlm.nih.gov/gene/?term=2864726) | 44 -- 54 | -7.84768 | fur2 | Iron response regulator |
| 0.0419571 | [BH10630](http://www.ncbi.nlm.nih.gov/gene/?term=2865814) | 138 -- 144 | -7.78656 | valS | valyl-tRNA synthetase |
| 0.0434967 | [BH05230](http://www.ncbi.nlm.nih.gov/gene/?term=2864947) | 27 -- 33 | -7.72823 |  | oxidoreductase |
| 0.0453431 | [BH05910](http://www.ncbi.nlm.nih.gov/gene/?term=2865543) | 118 -- 126 | -7.66077 | hupB | DNA-binding protein |
| 0.0459449 | [BH04570](http://www.ncbi.nlm.nih.gov/gene/?term=2865519) | 128 -- 135 | -7.63934 | ppdK | pyruvate phosphate dikinase |
| 0.0465521 | [BH07810](http://www.ncbi.nlm.nih.gov/gene/?term=2864570) | 22 -- 40 | -7.61798 | rpsI | 30S ribosomal protein S9 |
| 0.0465684 | [BH01460](http://www.ncbi.nlm.nih.gov/gene/?term=2864704) | 8 -- 22 | -7.61741 |  |  |
| 0.0470231 | [BH11320](http://www.ncbi.nlm.nih.gov/gene/?term=2865755) | 142 -- 148 | -7.60159 | mraW | S-adenosyl-methyltransferase MraW |
| 0.0494988 | [BH13360](http://www.ncbi.nlm.nih.gov/gene/?term=2865505) | 135 -- 145 | -7.51788 |  |  |
| 0.0505418 | [BH00240](http://www.ncbi.nlm.nih.gov/gene/?term=2865606) | 92 -- 102 | -7.48378 | lysK | lysyl-tRNA synthetase |
| 0.0508552 | [BH14550](http://www.ncbi.nlm.nih.gov/gene/?term=2865119) | 101 -- 125 | -7.47366 |  |  |
| 0.0521488 | [BH13590](http://www.ncbi.nlm.nih.gov/gene/?term=2864858) | 83 -- 98 | -7.4325 |  | transcriptional regulator |
| 0.0525275 | [BH02890](http://www.ncbi.nlm.nih.gov/gene/?term=2865801) | 44 -- 55 | -7.42063 |  |  |
| 0.0537225 | [BH02330](http://www.ncbi.nlm.nih.gov/gene/?term=2865246) | 119 -- 129 | -7.38369 |  |  |
| 0.0548603 | [BH05030](http://www.ncbi.nlm.nih.gov/gene/?term=2864561) | 130 -- 150 | -7.34922 | rpoZ | DNA-directed RNA polymerase subunit omega |
| 0.0550232 | [BH08380](http://www.ncbi.nlm.nih.gov/gene/?term=2865368) | 134 -- 148 | -7.34434 | murI | glutamate racemase |
| 0.0554307 | [BH05950](http://www.ncbi.nlm.nih.gov/gene/?term=2864731) | 12 -- 30 | -7.33219 | gatB | aspartyl/glutamyl-tRNA amidotransferase subunit B |
| 0.0556962 | [BH05350](http://www.ncbi.nlm.nih.gov/gene/?term=2865163) | 131 -- 149 | -7.32432 | fabG | 3-ketoacyl-ACP reductase |
| 0.0557229 | [BH02470](http://www.ncbi.nlm.nih.gov/gene/?term=2865449) | 54 -- 77 | -7.32353 | phoU | phosphate transporter PhoU |
| 0.0562777 | [BH15130](http://www.ncbi.nlm.nih.gov/gene/?term=2865746) | 138 -- 145 | -7.3072 |  |  |
| 0.0571938 | [BH04540](http://www.ncbi.nlm.nih.gov/gene/?term=2865373) | 141 -- 148 | -7.28056 | mutY | A/G-specific adenine glycosylase |
| 0.0574936 | [BH14150](http://www.ncbi.nlm.nih.gov/gene/?term=2864907) | 5 -- 12 | -7.27193 |  |  |
| 0.0588959 | [BH07910](http://www.ncbi.nlm.nih.gov/gene/?term=2865277) | 78 -- 88 | -7.2321 |  | helicase fragment |
| 0.0590895 | [BH10360](http://www.ncbi.nlm.nih.gov/gene/?term=2866004) | 114 -- 124 | -7.22667 | rplF | 50S ribosomal protein L6 |
| 0.0597713 | [BH08920](http://www.ncbi.nlm.nih.gov/gene/?term=2864977) | 138 -- 145 | -7.20768 | nuoD | NADH dehydrogenase subunit D |
| 0.0599651 | [BH02410](http://www.ncbi.nlm.nih.gov/gene/?term=2865622) | 7 -- 17 | -7.20232 | moeB | molybdopterin biosynthesis protein MoeB |
| 0.0601427 | [BH14650](http://www.ncbi.nlm.nih.gov/gene/?term=2865721) | 103 -- 125 | -7.19742 |  |  |
| 0.0608746 | [BH02530](http://www.ncbi.nlm.nih.gov/gene/?term=2864809) | 21 -- 47 | -7.17737 | rnd1 | ribonuclease d |
| 0.0615017 | [BH06360](http://www.ncbi.nlm.nih.gov/gene/?term=2864538) | 63 -- 77 | -7.16037 |  | ABC transporter permease |
| 0.0623885 | [BH08860](http://www.ncbi.nlm.nih.gov/gene/?term=2864983) | 124 -- 147 | -7.1366 | nuoJ | NADH dehydrogenase subunit J |
| 0.0637226 | [BH02020](http://www.ncbi.nlm.nih.gov/gene/?term=2865372) | 62 -- 75 | -7.10142 | mutT | Mutator mutT protein |
| 0.0638902 | [BH15740](http://www.ncbi.nlm.nih.gov/gene/?term=2864817) | 136 -- 147 | -7.09705 | trwF | trwF protein |
| 0.0641675 | [BH09260](http://www.ncbi.nlm.nih.gov/gene/?term=2864475) | 25 -- 31 | -7.08984 |  |  |
| 0.0646549 | [BH11430](http://www.ncbi.nlm.nih.gov/gene/?term=2865764) | 87 -- 101 | -7.07724 |  |  |
| 0.0652274 | [BH15560](http://www.ncbi.nlm.nih.gov/gene/?term=2865574) | 90 -- 108 | -7.06255 | korA | korA protein |
| 0.0673175 | [BH03110](http://www.ncbi.nlm.nih.gov/gene/?term=2865639) | 141 -- 149 | -7.00991 |  | Phage protein |
| 0.067384 | [BH02900](http://www.ncbi.nlm.nih.gov/gene/?term=2865802) | 18 -- 24 | -7.00826 |  | anti-repressor protein |
| 0.067432 | [BH02220](http://www.ncbi.nlm.nih.gov/gene/?term=2865592) | 108 -- 122 | -7.00707 | lnt | apolipoprotein N-acyltransferase |
| 0.0681261 | [BH13530](http://www.ncbi.nlm.nih.gov/gene/?term=2865623) | 51 -- 57 | -6.98995 | groEL | molecular chaperone GroEL |
| 0.0683043 | [BH00360](http://www.ncbi.nlm.nih.gov/gene/?term=2865966) | 90 -- 122 | -6.98558 |  | CysZ-like protein |
| 0.0683402 | [BH16290](http://www.ncbi.nlm.nih.gov/gene/?term=2865450) | 116 -- 122 | -6.9847 | plsC | 1-acyl-sn-glycerol-3-phosphate acyltransferase |
| 0.0698213 | [BH04210](http://www.ncbi.nlm.nih.gov/gene/?term=2865219) | 106 -- 113 | -6.9488 | thrB1 | 4-diphosphocytidyl-2-C-methyl-D-erythritol kinase |
| 0.0718559 | [BH10440](http://www.ncbi.nlm.nih.gov/gene/?term=2866012) | 130 -- 138 | -6.90061 | rplP | 50S ribosomal protein L16 |
| 0.0718778 | [BH11370](http://www.ncbi.nlm.nih.gov/gene/?term=2865759) | 144 -- 150 | -6.9001 |  | regulatory protein |
| 0.072549 | [BH10310](http://www.ncbi.nlm.nih.gov/gene/?term=2865181) | 129 -- 140 | -6.88448 | secY | preprotein translocase subunit SecY |
| 0.0727318 | [BH03820](http://www.ncbi.nlm.nih.gov/gene/?term=2864807) | 99 -- 116 | -6.88025 | rluD | ribosomal large subunit pseudouridine synthase D |
| 0.0733372 | [BH00120](http://www.ncbi.nlm.nih.gov/gene/?term=2865990) | 82 -- 94 | -6.86631 |  | SAM dependent methyltransferase |
| 0.0735253 | [BH06050](http://www.ncbi.nlm.nih.gov/gene/?term=2864990) | 68 -- 93 | -6.862 | nusG | transcription antitermination protein NusG |
| 0.0739643 | [BH15330](http://www.ncbi.nlm.nih.gov/gene/?term=2865395) | 13 -- 24 | -6.85198 | ATPG | ATP synthase F0F1 subunit gamma |
| 0.0757979 | [BH03150](http://www.ncbi.nlm.nih.gov/gene/?term=2865643) | 140 -- 150 | -6.81071 |  | virulence-associated protein |
| 0.0771194 | [BH08180](http://www.ncbi.nlm.nih.gov/gene/?term=2865292) | 1 -- 7 | -6.78153 |  |  |
| 0.0778074 | [BH07150](http://www.ncbi.nlm.nih.gov/gene/?term=2865177) | 92 -- 101 | -6.76652 | fhaB4 | filamentous hemagglutinin |
| 0.0784907 | [BH10370](http://www.ncbi.nlm.nih.gov/gene/?term=2864569) | 2 -- 25 | -6.75173 | rpsH | 30S ribosomal protein S8 |

**Table S5. mRNA targets for Brt1 predicted with RNAPredator algorithm**

| **Locus tag** | **mRNA start** | **mRNA end** | **sRNA start** | **sRNA end** | **Energy [kJ/mol]** | **Gene annotation** |
| --- | --- | --- | --- | --- | --- | --- |
| BH14360 | -173 | -149 | 1 | 25 | -23.21 | resolvase fragment like protein |
| BH16170 | 257 | 284 | 166 | 191 | -19.7 | diaminopimelate decarboxylase |
| BH11940 | 191 | 211 | 166 | 185 | -16.65 | regulatory protein |
| BH12730 | -97 | -69 | 165 | 194 | -16.45 | ornithine decarboxylase |
| BH12230 | 997 | 1007 | 166 | 176 | -15.51 | ABC transporter; periplasmic oligopeptide binding protein |
| BH10250 | 561 | 581 | 166 | 186 | -15.36 | Serine protease |
| BH12740 | 177 | 205 | 165 | 194 | -15.28 | hypothetical protein BH12740 |
| BH00460 | 1256 | 1281 | 167 | 191 | -14.88 | hypothetical protein BH00460 |
| BH04390 | 299 | 323 | 172 | 190 | -14.86 | hypothetical protein BH04390 |
| BH06020 | 360 | 370 | 166 | 176 | -14.7 | elongation factor Tu |
| BH10530 | 361 | 371 | 166 | 176 | -14.7 | elongation factor Tu |
| BH15330 | 555 | 581 | 166 | 186 | -14.25 | F0F1 ATP synthase subunit gamma |
| BH03280 | -116 | -96 | 3 | 25 | -14 | hypothetical protein BH03280 |
| BH00650 | 1362 | 1372 | 166 | 176 | -13.95 | molecular chaperone DnaK |
| BH01490 | -88 | -74 | 161 | 176 | -13.83 | adhesin |
| BH05220 | 465 | 475 | 166 | 176 | -13.81 | phosphatidylserine synthase |
| BH09540 | -82 | -70 | 164 | 176 | -13.35 | hypothetical protein BH09540 |
| BH11540 | 463 | 489 | 165 | 188 | -13.15 | DNA primase |
| BH07630 | 480 | 491 | 182 | 193 | -13.12 | 3 oxoacyl (acyl carrier protein) synthase III |
| BH07840 | 553 | 572 | 166 | 185 | -13.09 | DNA topoisomerase IV subunit B |
| BH02940 | 190 | 214 | 171 | 192 | -13.05 | hypothetical protein BH02940 |
| BH16570 | 811 | 823 | 164 | 176 | -13.04 | malate dehydrogenase |
| BH09240 | 430 | 459 | 166 | 194 | -12.84 | Phage related lysozyme |
| BH04850 | 371 | 394 | 104 | 122 | -12.81 | DNA polymerase |
| BH06720 | 336 | 356 | 104 | 124 | -12.73 | hypothetical protein BH06720 |
| BH03950 | 63 | 73 | 166 | 176 | -12.71 | hypothetical protein BH03950 |
| BH15040 | 545 | 573 | 165 | 191 | -12.68 | ABC transporter; ATP binding protein |
| BH09550 | 901 | 918 | 160 | 176 | -12.62 | permease protein |
| BH12170 | 404 | 414 | 165 | 175 | -12.59 | ABC transporter permease protein |
| BH12860 | 107 | 133 | 166 | 185 | -12.53 | hypothetical protein BH12860 |
| BH08610 | 600 | 608 | 166 | 174 | -12.42 | nitrogenase cofactor synthesis protein nifS |
| BH15310 | 311 | 320 | 185 | 194 | -12.38 | ATP synthase epsilon chain |
| BH14730 | 372 | 380 | 165 | 173 | -12.28 | hypothetical protein BH14730 |
| BH08750 | 2503 | 2521 | 165 | 188 | -12.22 | transcription repair coupling factor |
| BH06980 | 1067 | 1086 | 57 | 75 | -12.18 | hypothetical protein BH06980 |
| BH06990 | 61 | 80 | 57 | 75 | -12.1 | hypothetical protein BH06990 |
| BH16300 | 123 | 142 | 166 | 184 | -12.1 | penicillin binding protein |
| BH01930 | 114 | 136 | 166 | 188 | -12.08 | ribonucleotide reductase stimulatory prot |
| BH15360 | 687 | 701 | 160 | 174 | -12.06 | primosome assembly protein PriA |
| BH00730 | 456 | 482 | 171 | 194 | -12.02 | hypothetical protein BH00730 |
| BH05050 | 241 | 262 | 166 | 182 | -12.01 | orotate phosphoribosyltransferase |
| BH07150 | 5512 | 5525 | 182 | 194 | -12.01 | filamentous hemagglutinin |
| BH06670 | 5503 | 5516 | 182 | 194 | -12.01 | filamentous hemagglutinin |
| BH04040 | 457 | 476 | 166 | 184 | -11.94 | Na+/H antiporter |
| BH07250 | 336 | 356 | 104 | 124 | -11.92 | hypothetical protein BH07250 |
| BH15300 | -129 | -120 | 185 | 194 | -11.81 | transport protein transmembrane |
| BH15810 | 211 | 237 | 165 | 193 | -11.79 | 50S ribosomal protein L19 |
| BH02080 | -154 | -139 | 179 | 193 | -11.76 | ATP dependent protease ATP binding sub |
| BH04770 | 570 | 597 | 170 | 193 | -11.76 | Serine protease |
| BH07470 | 1645 | 1661 | 177 | 190 | -11.75 | malic enzyme |
| BH01240 | 624 | 634 | 166 | 176 | -11.75 | cytochrome o ubiquinol oxidase subunit |
| BH02440 | 891 | 907 | 166 | 184 | -11.72 | ABC transporter permease protein |
| BH12180 | 650 | 664 | 162 | 176 | -11.71 | ABC transporter; periplasmic oligopeptide binding protein |
| BH09750 | -95 | -85 | 165 | 176 | -11.63 | hypothetical protein BH09750 |
| BH00280 | 2274 | 2296 | 165 | 191 | -11.63 | hypothetical protein BH00280 |
| BH05460 | 170 | 193 | 103 | 122 | -11.58 | DNA polymerase III subunit chi |
| BH13810 | 1112 | 1121 | 165 | 174 | -11.56 | hypothetical protein BH13810 |
| BH12360 | -110 | -96 | 162 | 176 | -11.56 | hypothetical protein BH12360 |
| BH10980 | 517 | 525 | 165 | 173 | -11.5 | thymidylate synthase |
| BH02580 | 1536 | 1544 | 165 | 173 | -11.5 | isoleucyl tRNA synthetase |
| BH02690 | 183 | 191 | 166 | 174 | -11.44 | DNA mismatch repair protein |
| BH07180 | -46 | -25 | 103 | 124 | -11.43 | hypothetical protein BH07180 |
| BH02710 | 582 | 596 | 162 | 176 | -11.39 | L lactate dehydrogenase |
| BH16450 | -116 | -94 | 172 | 194 | -11.32 | putative monovalent cation/H+ antiporter subunit C |
| BH13030 | 4183 | 4196 | 93 | 106 | -11.3 | surface protein |
| BH11180 | 394 | 405 | 165 | 176 | -11.29 | cell division protein FtsZ |
| BH09960 | 101 | 110 | 164 | 173 | -11.25 | hypothetical protein BH09960 |
| BH00030 | 54 | 64 | 166 | 176 | -11.24 | shikimate 5 dehydrogenase |
| BH02070 | 428 | 443 | 179 | 193 | -11.23 | ATP dependent protease peptidase subunit |
| BH05790 | -33 | -24 | 166 | 175 | -11.23 | lipoyl synthase |
| BH08520 | -173 | -165 | 166 | 174 | -11.22 | enoyl CoA hydratase |
| BH11070 | 1917 | 1930 | 93 | 107 | -11.22 | DNA helicase II |
| BH07650 | -177 | -167 | 166 | 176 | -11.2 | hypothetical protein BH07650 |
| BH05210 | 100 | 129 | 166 | 194 | -11.19 | phosphatidylserine decarboxylase |
| BH09740 | 2179 | 2189 | 165 | 176 | -11.17 | phosphoribosylformylglycinamidine synth |
| BH11090 | 405 | 422 | 105 | 121 | -11.17 | sco1/2 family protein |
| BH14520 | 467 | 488 | 170 | 191 | -11.16 | phage related protein |
| BH10060 | 1357 | 1366 | 166 | 175 | -11.15 | transport transmembrane protein |
| BH02900 | -57 | -52 | 181 | 186 | -11.12 | anti repressor protein |
| BH12720 | 503 | 522 | 165 | 182 | -11.04 | acetyltransferase |
| BH00200 | 138 | 145 | 166 | 173 | -11.04 | hypothetical protein BH00200 |
| BH06420 | 90 | 101 | 182 | 193 | -11.03 | hypothetical protein BH06420 |
| BH11190 | 1117 | 1125 | 165 | 173 | -11.03 | cell division protein ftsA |
| BH13590 | 1 | 12 | 165 | 176 | -10.98 | transcriptional regulator |
| BH03440 | -56 | -51 | 181 | 186 | -10.98 | anti repressor protein |
| BH12280 | 512 | 520 | 165 | 173 | -10.96 | hypothetical protein BH12280 |
| BH16160 | 158 | 169 | 164 | 175 | -10.95 | hypothetical protein BH16160 |
| BH08620 | 1135 | 1142 | 166 | 173 | -10.92 | hypothetical protein BH08620 |
| BH08240 | 796 | 805 | 166 | 175 | -10.91 | aspartyl/glutamyl tRNA amidotransferase subunit A |
| BH15460 | 1639 | 1655 | 161 | 176 | -10.9 | ABC transporter; ATP binding protein |
| BH08840 | 1192 | 1206 | 162 | 176 | -10.9 | NADH dehydrogenase subunit L |
| BH00700 | 130 | 157 | 165 | 190 | -10.89 | hypothetical protein BH00700 |
| BH00320 | 354 | 362 | 166 | 174 | -10.87 | NADH ubiquinone oxidoreductase |
| BH00610 | 162 | 181 | 164 | 191 | -10.86 | two component sensor histidine kinase |
| BH11830 | 345 | 362 | 174 | 190 | -10.83 | ribose phosphate pyrophosphokinase |
| BH02630 | 455 | 476 | 50 | 72 | -10.79 | pmbA protein, mccb17 |
| BH01560 | 210 | 223 | 94 | 107 | -10.79 | GTPase ObgE |
| BH12330 | -163 | -152 | 165 | 176 | -10.78 | pyrroline 5 carboxylate reductase |
| BH07920 | 756 | 766 | 166 | 176 | -10.76 | hemolysin activation protein hec |
| BH07820 | 414 | 432 | 171 | 189 | -10.74 | 50S ribosomal protein L13 |

**A B**

**Figure S2.** Growth Curves of the B. henselae strains used in this study. **A**. Strains *Bhh*13, *Bhh*72 and *Bhh*73 Grown in liquid Schneider’s medium with 50 µg/ml kanamycin. **B.** Houston-1 strain of *B. henselae* (*Bh*) and the ∆*badA* mutant (*Bhh*17) grown in Schneider’s medium with no antibiotics.

**Table S6. Oligonucleotide primers/probes used in this study.**

| \| **Oligonucleotide** \| **Sequence (5’ to 3’)** \| **Purpose** \| \| --- \| --- \| --- \| \| BrtF \| TCGCATACGCAACGACCAATA \| Forward PCR primer to detect all Brt RNAs \| \| BrtR \| TGGACATAGCAAAATCTCCCGGAA \| Reverse PCR primer to detect all Brt RNAs \| \| ASBrt \| CCATGCATCATCATCATCATCATG \| Forward PCR primer to detect antisense Brt1 with BrtF \| \| BadAF \| CGTTACCGGTGGTCAACTCT \| Forward PCR primer to detect *badA* (BH01510) mRNA \| \| BadAR \| CCAGTCAAAGCTTCCGCTAC \| Reverse PCR primer to detect *badA* mRNA \| \| Trp1F \| CTCCCATTTAGGCATAACTTTC \| Forward PCR primer to detect *trp1* mRNA (BH12870) \| \| Trp1R \| GATATTCTGTCGTTATCAGGGTAG \| Reverse PCR primer to detect *trp1* mRNA \| \| RplDF \| ATGCGCATGACTACGAACAA \| Forward PCR primer for reference gene *rplD* mRNA \| \| RplDR \| CAGGGGCGAAGATGTTTAAG \| Reverse PCR primer for reference gene *rplD* mRNA \| \| BrtProbe \| CCCGGAATACCAATGCGAGGGCGCTCGCA \| Northern blot probe to detect all Brt RNAs \| \| asBrtProbe \| GCTCTAACATCGAACACAGAATAAATCCGCTTTGC \| Northern blot probe to detect anti-sense Brt1 \| \| Brt1asF \| GTTATGGATCCGGTTGCGAGCGCCCTCGCATTGG  *Bam*HI* \| Forward PCR primer to amplify the Brt1 coding region for directional cloning into pNS2Trc to generate anti-sense Brt1 RNA \| \| Brt1asR \| GTTATTCTAGATGTTTTGGTCGCATACGCAACG  *Xba*I* \| Reverse PCR primer to amplify the Brt1 coding region for directional cloning into pNS2Trc to generate anti-sense Brt1 RNA \| \| Trp1F \| CGGCGGATCCCGAACCAAAAATCCAC  *Bam*HI* \| Forward PCR primer to amplify *trps* for directional cloning into pNS2Trc \| \| Trp1R \| CGGCTCTAGAAGGTAGAGGCTTGTGATGTG  *Xba*I* \| Reverse PCR primer to amplify *trps* for directional cloning into pNS2Trc \|   *Restriction endonuclease sites added to the 5’ end of the primer to facilitate cloning |
| --- | --- | --- | --- | --- | --- | --- | --- | --- | --- | --- | --- | --- | --- | --- | --- | --- | --- | --- | --- | --- | --- | --- | --- | --- | --- | --- | --- | --- | --- | --- | --- | --- | --- | --- | --- | --- | --- | --- | --- | --- | --- | --- | --- | --- | --- | --- | --- | --- |
